# Development and Validation of an MRI‐Based Radiomics Nomogram to Predict the Prognosis of De Novo Oligometastatic Prostate Cancer Patients

**DOI:** 10.1002/cam4.70481

**Published:** 2024-12-20

**Authors:** Wen‐Qi Liu, Yu‐Ting Xue, Xu‐Yun Huang, Bin Lin, Xiao‐Dong Li, Zhi‐Bin Ke, Dong‐Ning Chen, Jia‐Yin Chen, Yong Wei, Qing‐Shui Zheng, Xue‐Yi Xue, Ning Xu

**Affiliations:** ^1^ Department of Urology, The First Affiliated Hospital Fujian Medical University Fuzhou China; ^2^ Department of Urology, National Regional Medical Center, Binhai Campus of the First Affiliated Hospital Fujian Medical University Fuzhou China; ^3^ Fujian Key Laboratory of Precision Medicine for Cancer, The First Affiliated Hospital Fujian Medical University Fuzhou China

**Keywords:** nomogram, oligometastatic prostate cancer (PCa), overall survival (OS), radiomics

## Abstract

**Objective:**

We aimed to develop and validate a nomogram based on MRI radiomics to predict overall survival (OS) for patients with de novo oligometastatic prostate cancer (PCa).

**Methods:**

A total of 165 patients with de novo oligometastatic PCa were included in the study (training cohort, *n* = 115; validating cohort, *n* = 50). Among them, MRI scans were conducted and T2‐weighted imaging (T2WI) and apparent diffusion coefficient (ADC) sequences were collected for radiomics features along with their clinicopathological features. Radiological features were extracted from T2WI and ADC sequences for prostate tumors. Univariate Cox regression analysis and the least absolute shrinkage and selection operator (LASSO) combined with 10‐fold cross‐validation were used to select the optimal features on each sequence. Then, a weighted radiomics score (Rad‐score) was generated and independent risk factors were obtained from univariate and multivariate Cox regressions to build the nomogram. Model performance was assessed using receiver operating characteristic (ROC) curves, calibration, and decision curve analysis (DCA).

**Results:**

Eastern Cooperative Oncology Group (ECOG) score, absolute neutrophil count (ANC) and Rad‐score were included in the nomogram as independent risk factors for OS in de novo oligometastatic PCa patients. We found that the areas under the curves (AUCs) in the training cohort were 0.734, 0.851, and 0.773 for predicting OS at 1, 2, and 3 years, respectively. In the validating cohort, the AUCs were 0.703, 0.799, and 0.833 for predicting OS at 1, 2, and 3 years, respectively. Furthermore, the clinical relevance of the predictive nomogram was confirmed through the analysis of DCA and calibration curve analysis.

**Conclusion:**

The MRI‐based nomogram incorporating Rad‐score and clinical data was developed to guide the OS assessment of oligometastatic PCa. This helps in understanding the prognosis and improves the shared decision‐making process.

## Graphical abstract

Illustration of radiomics study framework. We first extracted 1446 quantitative radiomic features from masked PCa T2‐weighted imaging and diffusion‐weighted imaging data. Next, univariate Cox regression analysis and the least absolute shrinkage and selection operator (LASSO) combined with 10‐fold cross‐validation were used to select the optimal features on each sequence. Then, a weighted radiomics score (Rad‐score) was generated and independent risk factors were obtained from univariate and multivariate Cox regressions to build the nomogram. Model performance was assessed using receiver operating characteristic (ROC) curves, calibration, and decision curve analysis.

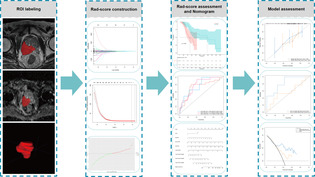

## 1 Introduction

Prostate cancer (PCa) imposes a significant global burden; it ranks as the second most frequently diagnosed cancer in men globally and accounted for 375,304 deaths in 2020 [1]. Due to widespread prostate‐specific antigen (PSA) screening, most patients with PCa are detected through elevated PSA levels and the majority are in the early stages of the disease [2]. However, there are still some patients who seek medical attention because of clinical symptoms such as difficulty in urination and generalized pain, and these patients have a high probability of having developed distant metastases by the time of their visit to the doctor. Even though the 5‐year survival rate of PCa is as high as 98.2%, once distant metastasis occurs, the patient's 5‐year survival rate is reduced by 30% [3]. Fortunately, as more and more approved new drugs become available, the overall survival (OS) of metastatic patients has improved dramatically [4, 5, 6, 7, 8].

Patients with advanced PCa may display either widely metastatic or oligometastatic conditions. While the exact definition oligometastatic of PCa remains somewhat elusive, the clinical relevance of local treatment for de novo oligometastatic PCa has recently emerged as a topic of significant interest [9, 10]. Certain patients with this condition have the potential to be cured or experience benefits from aggressive local therapy [11]. Increasing clinical data indicates that individuals with oligometastatic disease exhibit better clinical responses when undergoing therapy targeted at their metastases [12]. In addition, the randomized controlled trial (ORIOLE) has shown that radiotherapy (RT) improves median progression‐free survival (PFS) of oligometastatic PCa [13]. Although patients with oligometastases can benefit from local therapy in certain situations, it is not clear whether all patients with oligometastases benefit from local therapy. Therefore, androgen deprivation therapy (ADT) remains the treatment of choice for patients with de novo oligometastases. Furthermore, in a study led by Junryo Rii and colleagues, it was discovered that nonregional lymph node metastases, Gleason Grade 5, and three or more bone metastases are independent prognostic factors associated with a diminished OS in de novo oligometastatic PCa. These findings enabled the classification of patients into low‐risk, intermediate‐risk, and high‐risk groups based on the number of these risk factors, facilitating a valuable stratification of both OS and PFS [14]. However, this method only provides a rough grouping of patients and does not individualize the prognosis for those with oligometastases. Hence, there is a continued need for a more individualized yet comprehensive approach to accurately predict the prognosis of oligometastatic PCa, with the ultimate goal of enhancing the quality of patient care.

Radiomics has emerged as a cutting‐edge concept at the intersection of computer science and medicine. This innovative approach utilizes intricate mathematical algorithms to conduct in‐depth analysis and extract valuable medical imaging data from various modalities such as CT scans, MRIs, and PET scans [15]. Radiomics features have made substantial advancements in evaluating the effectiveness of treatments and predicting prognosis in various cancers [16, 17, 18, 19]. It also offers valuable information that can be applied to the detection, risk assessment, and treatment planning for PCa, and most of these features were based on multiparametric magnetic resonance imaging (mp‐MRI) images [20, 21, 22]. To the best of our knowledge, there is currently no published literature that has explored whether a radiomics approach based on mp‐MRI could enhance the prediction of OS in oligometastatic PCa patients.

Consequently, the aim of this study is to develop a robust clinical nomogram that incorporates the radiomics signature (R‐signature) derived from mp‐MRI and other clinically relevant prognostic indicators for individuals initially diagnosed with oligometastatic PCa. The purpose is to create a tool that can predict their survival outcomes and, in turn, assist in tailoring individualized treatment strategies.

## 2 Patients and Methods

### 2.1 Patient Selection

**FIGURE 1:**
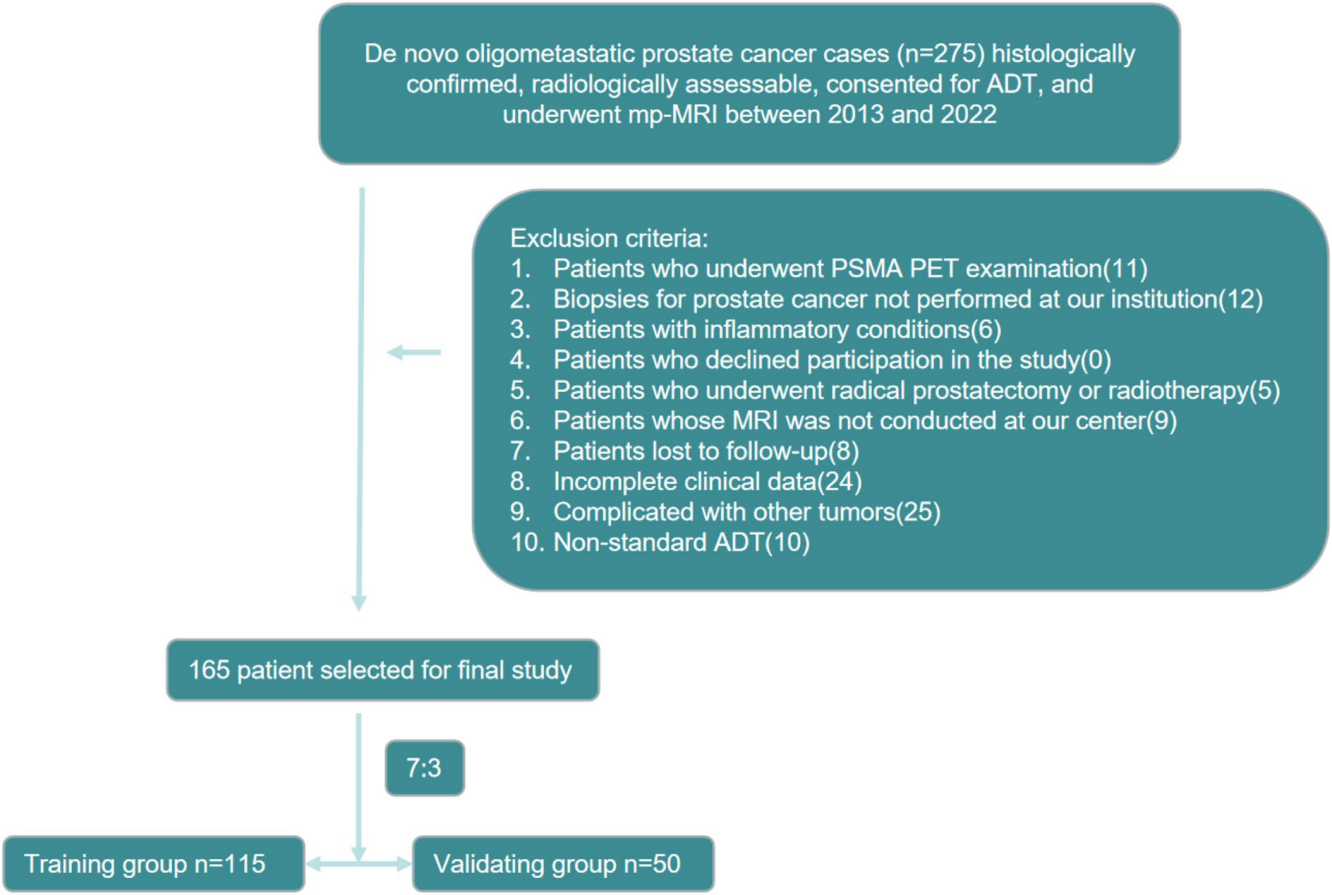
Patient selection flow chart. Includes exclusion criteria and grouping.

This study received approval from the Ethics Committee of the First Affiliated Hospital of Fujian Medical University (Approved No. of Ethic Committee: MTCA and ECFAH of FMU [2024]646). A retrospective analysis was conducted on individuals with histologically confirmed and radiologically assessable oligometastatic PCa who initiated ADT (using luteinizing hormone‐releasing hormone analogs, including leuprolide, goserelin, triptorelin, and histrelin), during the period from 2019 to 2022. Based on previous studies, this study defined oligometastatic PCa as having only lymph node and bone metastases with less than or equal to five metastases [23, 24, 25, 26, 27]. All patients underwent both computed tomography and a ^99m^technetium methylene diphosphate (^99m^Tc‐MDP) bone scan. Skilled radiologists utilized the Union for International Cancer Control classifications to establish the TNM stage and also evaluated the presence and quantity of lymph nodes and bone metastases. mp‐MRI scans were collected from the Picture Archiving and Communication System (PACS) managed by GE. The patient grouping is illustrated in Figure 1. Inclusion criteria: (1) Histologically confirmed and radiologically assessable de novo oligometastatic PCa cases who underwent mp‐MRI; (2) confirmation of de novo oligometastatic PCa via conventional imaging (prostate MRI and bone scan); (3) patients consenting to ADT at our center; (4) patients who agreed to participate in the study and provided written informed consent; and (5) availability of complete clinicopathologic data. Exclusion criteria: (1) Patients who underwent PSMA PET examination; (2) biopsy for PCa was not performed at our hospital; (3) patients with inflammatory conditions (e.g., severe urinary tract infections, pneumonia, and sepsis) that may affect absolute neutrophil count (ANC) and neutrophil–lymphocyte ratio (NLR); (4) patients who disagreed to be included in this study; (5) patients who underwent radical prostatectomy or RT; (6) patients whose MRI was not conducted at our center; (7) lost to follow‐up patients; (8) incomplete clinical data; (9) complicated with other tumors; and (10) patients receiving nonstandard ADT (including but not limited to intermittent ADT, different dosing schedules or frequencies of LHRH agonists/antagonists, or combined with other forms of endocrine therapy). With this, a total of 165 patients were included in the study. They were randomly allocated into two cohorts: 115 patients (70%) were assigned to the training cohort, and 50 patients (30%) were assigned to the validation cohort. This random allocation was performed using a simple randomization method without stratification, ensuring an unbiased division of patients between the two cohorts.

### 2.2 Clinical and Pathological Data

Clinical and pathological data were collected for analysis, including age, body mass index (BMI), ANC, absolute lymphocyte count (ALC), baseline NLR, PI‐RADS score, clinical T stage, lymph node involvement, International Society of Urological Pathology (ISUP) grading group [28], Eastern Cooperative Oncology Group (ECOG) score [29], bone metastases, and received additional chemotherapy or not. The ANC and ALC were determined using the BC‐6800Plus_3 instrument and accompanying reagents and were expressed in 10^9^ cells per liter (cells/L). The normal range for ANC is 1.8–6.0 × 10^9^ cells/L, and for ALC is 1.1–3.2 × 10^9^ cells/L. The endpoint of the study was OS, defined as time from ADT start to death from any cause or censored at the last follow‐up date.

### 2.3 MRI Examination

All patients underwent magnetic resonance imaging (MRI) examinations using a 3 Tesla magnet, specifically the SIEMENS Verio 3.0 T model. The imaging sequences employed included the following: Axial T2‐weighted imaging (T2WI) with the following parameters: repetition time (TR) of 3500 ms, echo time (TE) of 101 ms, slice thickness (ST) of 3 mm, no slice gap, field of view (FOV) measuring 200 × 200 mm, matrix size of 256 × 256, parallel imaging factor of 2, and signal averaging over three scans. T1‐weighted imaging (T1WI) with the following parameters: TR of 700 ms, TE of 11 ms, ST of 3 mm, no slice gap, FOV of 200 × 200 mm, matrix size of 192 × 116, parallel imaging factor of 2, and signal averaging over two scans. Diffusion‐weighted imaging (DWI) with the following parameters: TR of 5600 ms, TE of 93 ms, ST of 3.6 mm, no slice gap, FOV measuring 190 × 260 millimeters, matrix size of 160 × 102, parallel imaging factor of 2, and signal averaging over 10 scans. Two *b*‐values were used, namely, 50 and 800 s/mm^2^. Apparent diffusion coefficient (ADC) maps were generated from the directly acquired DW images using the specified *b*‐values of 50 and 800 s/mm^2^, following the guidelines of the European Society of Urogenital Radiology [30].

### 2.4 Image Segmentation, Data Extraction, and Radiomic Feature Extraction and Selection

**FIGURE 2:**
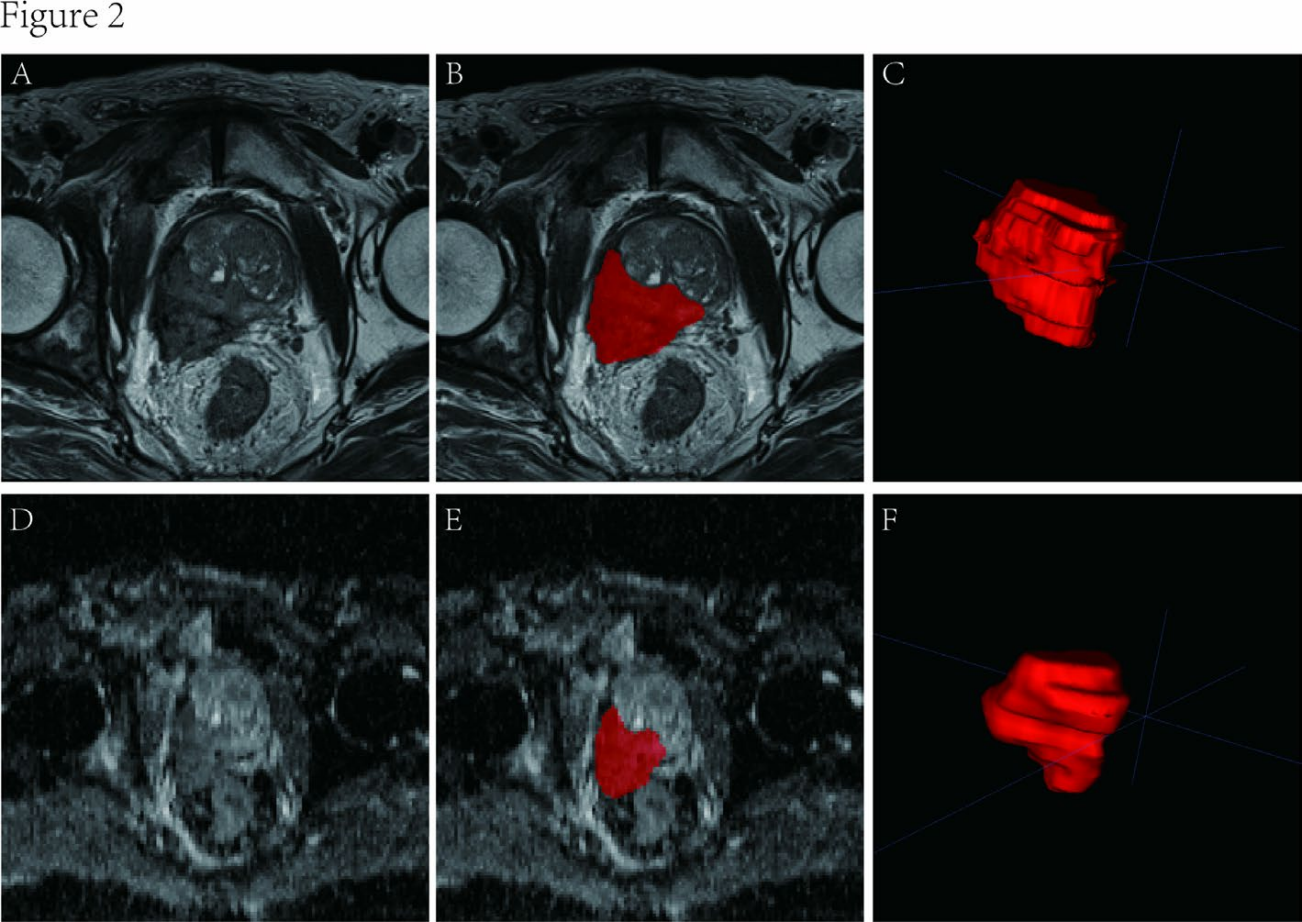
Schematic diagram of the prostate cancer ROI outline. (A) is the T2WI sequence with PCa in the right peripheral band, (B) is the T2WI sequence with ROI outline of PCa, (C) is the generated ROI of PCa in T2WI sequence, (D) is the ADC sequence with the cancer foci showing low signal, (E) is the ADC with ROI outline of PCa and (F) is the generated ROI of PCa in ADC sequence.

Two experienced radiologists in urological imaging, each with at least 5 years of expertise, employed the open‐source software ITK‐SNAP (www.itksnap.org) for the purpose of manually outlining regions of interest (ROIs) based on T2WI and ADC data [31]. These delineations were made along the contours of T2WI and ADC‐visible tumor regions, and both radiologists performed this task without prior knowledge of the histopathological results. Figure 2 illustrates the ROI diagrams for PCa.

In this research, radiomics characteristics were derived from both T2WI and ADC images. T2WI is effective in presenting the anatomical attributes of tumors and the surrounding periprostatic adipose tissue. Furthermore, it can provide insights into the potential involvement of peripheral nerves and seminal vesicles in PCa patients. T2WI also offers a wealth of valuable texture features. On the other hand, the ADC value offers an objective measure of the extent of water molecule diffusion within biological tissues, and it is closely associated with the tumor's malignancy. This attribute is particularly valuable as it helps to circumvent the penetration effect typically observed in tissues due to the prolonged T2 decay time in DWI [32]. This is consistent with most previous radiomics studies for detecting PCa lesions and treatment planning of PCa [20, 21, 22].

We utilized the open‐source tool Pyradiomics to extract radiomic features from each ROI in both the T2WI and ADC images. These extracted features encompassed various categories, including first‐order statistics, shape‐based (2D and 3D), gray level co‐occurrence matrix (GLCM), gray level run length matrix (GLRLM), gray level size zone matrix (GLSZM), gray level dependence matrix (GLDM), and neighboring gray‐tone difference matrix (NGTDM). In addition to these feature classes, there were also optional built‐in filters that could be applied, enhancing the versatility of the radiomic analysis. These optional filters included: Laplacian of Gaussian (LoG, based on SimpleITK functionality), wavelet (using the PyWavelets package), square, square root, logarithm, exponential, gradient (magnitude), and local binary pattern (LBP) 2D/3D [33]. A total of 1446 radiomic features were extracted from each ROI.

In the training cohort samples, the texture features were subjected to analysis through univariate Cox regression to initially identify features associated with OS based on a significance level of *p* < 0.05. Subsequently, a more comprehensive survival analysis was conducted using least absolute shrinkage and selection operator (LASSO)–Cox regression to further pinpoint radiomic features that are strongly linked to OS. To create a more refined predictive model, a radiomics score (Rad‐score) was formulated by taking into account the radiomic features that were retained after LASSO–Cox regression. These features were assigned weights determined through linear combination calculations. Based on the resulting median Rad‐score, the cases were divided into two distinct groups: a high‐risk group and a low‐risk group. Kaplan–Meier survival curves were then generated for each group, with corresponding Rad‐scores displayed alongside. To evaluate the statistical significance of the observed survival differences between these groups, log‐rank tests were employed. Additionally, the predictive value of OS was assessed using receiver operating characteristic (ROC) curves.

Univariate Cox regression was conducted to examine both clinicopathological variables and the Rad‐score in order to identify significant risk factors (with a significance threshold of *p* < 0.05). Variables that exhibited statistical significance were then subjected to further analysis using multivariate stepwise Cox regression to identify independent risk factors.

### 2.5 Intra‐ and Interobserver Consistency

The study utilized intraclass correlation coefficients (ICCs) to evaluate the consistency of radiomic feature extraction within and between observers, focusing on the training dataset. ICC values falling within the range 0.95–1.00 were deemed to indicate nearly perfect agreement, while values in the 0.75–0.95 range indicated substantial agreement. A range of 0.50–0.75 indicated moderate agreement, and values from 0 to 0.50 signified poor or inconsistent agreement [34]. Initially, both Radiologist 1 and Radiologist 2 randomly selected imaging data from 40 patients in the training set for the segmentation of ROIs and subsequent feature extraction. Following this, Radiologist 1 repeated this process 2 weeks later. ICCs were then computed to assess the degree of agreement between the features extracted by Radiologist 1 and Radiologist 2 from the ROIs, as well as between the features extracted by Radiologist 1 during the two separate sessions. As part of our data quality assurance, radiomic features displaying poor repeatability, defined as having an ICC of 0.75 or lower, were excluded from the subsequent analysis to ensure the reliability and consistency of our radiomic dataset.

### 2.6 Nomogram Construction and Performance Assessment

We leveraged the radiomic features along with the independent risk factors to create a nomogram tailored for patients with oligometastatic PCa. This nomogram was primarily founded on the mp‐MRI Rad‐score. To gauge the clinical utility and predictive performance of the nomogram, we employed several evaluation methods, including decision curve analysis (DCA), calibration curves, and ROC curves.

### 2.7 Statistical Analyses

Throughout this study, statistical analysis was performed using R software, specifically version 3.4.2. To determine statistical significance, a two‐sided significance level of *p* < 0.05 was employed, where values below this threshold were considered statistically significant.

## 3 Results

### 3.1 Patient Characteristics

A total of 165 histologically confirmed and radiologically assessable oligometastatic PCa patients were enrolled in this study. The statistical description of basic data of the enrolled patients is listed in Table 1. All patients were histologically confirmed and radiologically assessable oligometastatic PCa. No statistical differences were seen between the training cohort and validation cohort for any variables (*p* > 0.05).

**TABLE 1:** The baseline characteristics of patients with oligometastatic prostate cancer in the training and validating cohorts.

Variables	Total (*n* = 165)	Training (*n* = 115)	Validating (*n* = 50)	*p*
Age, median (Q1, Q3)	71.0 (65.0, 78.0)	71.0 (65.5, 78.0)	72.0 (64.5, 79.0)	0.482
BMI, median (Q1, Q3)	23.4 (21.0, 24.4)	23.4 (21.3, 24.4)	23.9 (21.0, 24.4)	0.836
Baseline NLR, median (Q1, Q3)	2.8 (2.0, 4.2)	2.7 (1.9, 3.7)	3.0 (2.0, 5.5)	0.138
ANC, median (Q1, Q3)	3.9 (2.9, 5.0)	3.9 (2.8, 4.7)	4.1 (3.1, 5.4)	0.19
ALC, median (Q1, Q3)	1.4 (1.1, 1.8)	1.4 (1.1, 1.8)	1.4 (1.0, 1.8)	0.438
PSA as diagnosis, *n* (%)				0.829
< 100	78 (47.3%)	55 (47.8%)	23 (46.0%)	
≥ 100	87 (52.7%)	60 (52.2%)	27 (54.0%)	
PI‐RADS score, *n* (%)				0.781
3	9 (5.5%)	7 (6.1%)	2 (4.0%)	
4	15 (9.1%)	9 (7.8%)	6 (12.0%)	
5	141 (85.5%)	99 (86.1%)	42 (84.0%)	
Clinical T stage, *n* (%)				0.948
T2	29 (17.6%)	19 (16.5%)	10 (20.0%)	
T3a	6 (3.6%)	5 (4.3%)	1 (2.0%)	
T3b	50 (30.3%)	36 (31.3%)	14 (28.0%)	
T4	80 (48.5%)	55 (47.8%)	25 (50.0%)	
Lymph node involvement, *n* (%)				0.302
N0	66 (40.0%)	43 (37.4%)	23 (46.0%)	
N+	99 (60.0%)	72 (62.6%)	27 (54.0%)	
ISUP grading group, *n* (%)				0.964
≤ 4	40 (24.2%)	28 (24.3%)	12 (24.0%)	
5	125 (75.8%)	87 (75.7%)	38 (76.0%)	
ECOG score, *n* (%)				0.258
0–1	134 (81.2%)	96 (83.5%)	38 (76.0%)	
2	31 (18.8%)	19 (16.5%)	12 (24.0%)	
Bone metastases, *n* (%)				0.743
No	22 (13.3%)	16 (13.9%)	6 (12.0%)	
Yes	143 (86.7%)	99 (86.1%)	44 (88.0%)	
Chemotherapy, *n* (%)				0.267
No	119 (72.1%)	80 (69.6%)	39 (78.0%)	
Yes	46 (27.9%)	35 (30.4%)	11 (22.0%)	

Abbreviations: ALC = absolute lymphocyte count, ANC = absolute neutrophil count, BMI = body mass index, ECOG score = Eastern Cooperative Oncology Group score, ISUP grading group = International Society of Urological Pathology grading group, NLR = neutral–lymphocyte ratio.

### 3.2 Laboratory Findings

The median ANC among the patients was 3.9 × 10^9^ cells/μL, with a range from 1.4 × 10^9^ cells/L to 12.76 × 10^9^ cells/L as depicted in Figure S1A. Figure S1B illustrates higher ANC values (above the median) were associated with better OS, whereas lower ANC values (below the median) were associated with poorer survival (*p* < 0.05). The standard deviations above and below the median also showed a significant correlation with OS.

### 3.3 Features Selection and Rad‐Score Construction

Our initial step involved screening the radiomics features associated with OS using a univariate Cox regression analysis, focusing on radiomic features extracted from the training cohort. Applying a statistical significance threshold of *p* < 0.05, we identified a total of 358 features that exhibited associations with OS (Table S1). Furthermore, to refine the selection process and pinpoint features with nonzero coefficients for inclusion, we employed LASSO–Cox regression, and a total of five radiomic features (including four T2WI features and one ADC feature) were selected. The details of LASSO–Cox regression are described in Figure S2. This technique enabled us to construct Rad‐score equations by incorporating the relevant features and their corresponding coefficients, as outlined below: Rad‐Score = wavelet‐LHL_firstorder_Kurtosis_T2WI * 0.0139524081666556 + wavelet‐LHL_glcm_Idn_T2WI * 4.84409032721217 + wavelet‐HLH_glcm_Imc1_T2WI * 1.53680433480562 + original_shape_Sphericity_ADC * (−0.257969157699035).

### 3.4 Radiomics Features Assessment

**FIGURE 3:**
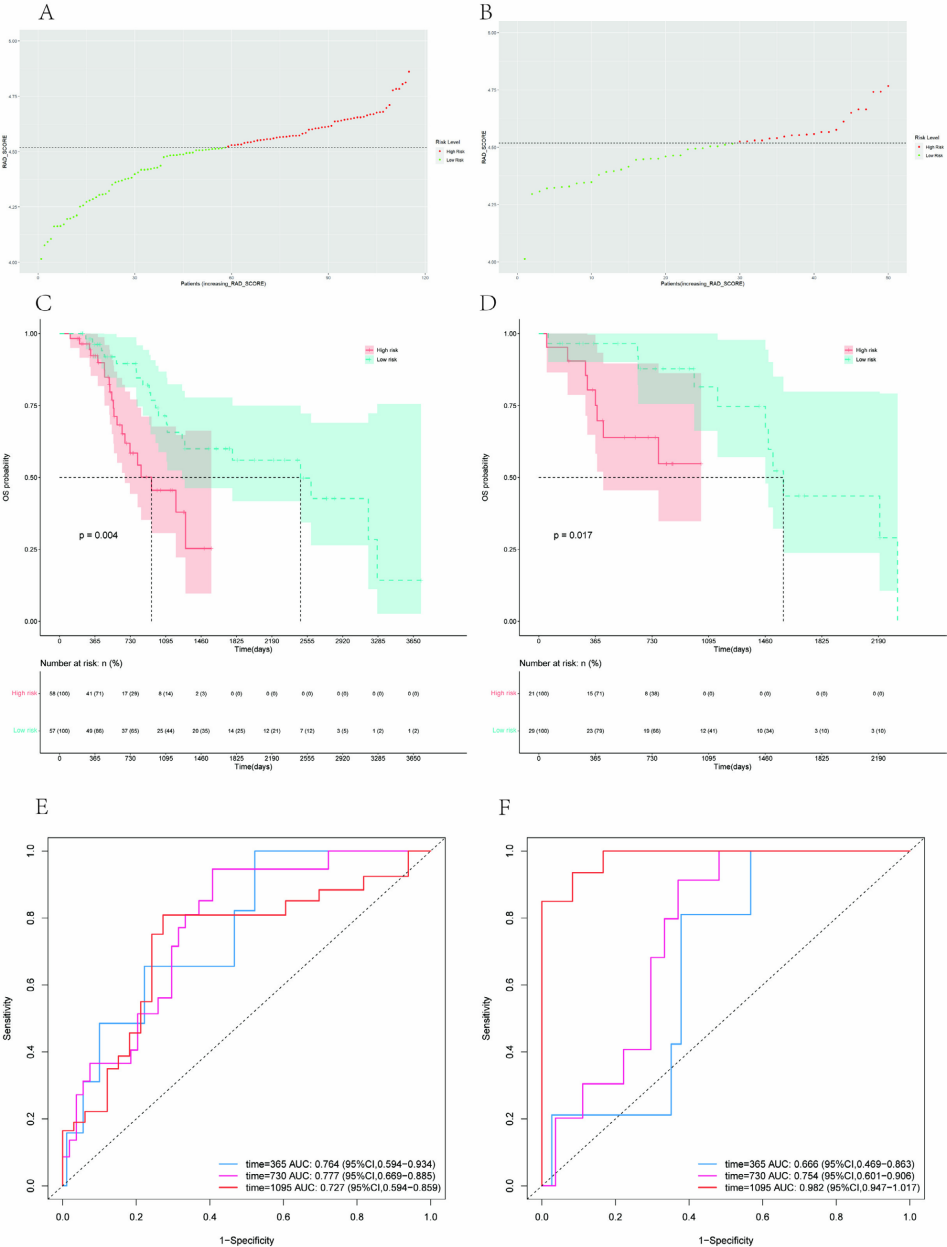
Rad‐score analysis of patients in the training and validation cohorts. Risk chart of the training cohort (A) and validating cohort (B). Rad‐score measured by Kaplan–Meier survival curves of the training cohort (C) and validating cohort (D), the log‐rank test was used to calculate *p* values, *p* < 0.05, and the differences were significant, both cohorts were divided into high‐risk and low‐risk groups. The ROC curves of the training cohort (E) and validation cohort (F) to predict the 1‐OS, 2‐OS, and 3‐OS.

The training cohort was divided into two groups based on the median Rad‐score, as depicted in Figure 3A,B. A higher Rad‐score indicates an increased risk and poorer prognosis. The Kaplan–Meier survival curves in Figure 3C demonstrate a statistically significant difference in OS (*p* = 0.004) between the two groups of the training cohort. Similar findings were observed in Figure 3D (*p* = 0.017). The ROC curves presented in Figure 3E illustrate the ability of Rad‐scores to predict OS at 1, 2, and 3 years, with corresponding AUC values of 0.764, 0.777, and 0.727. The validating cohort results, shown in Figure 3F, had AUC values of 0.666, 0.754, and 0.982, respectively.

### 3.5 Nomogram Construction

**FIGURE 4:**
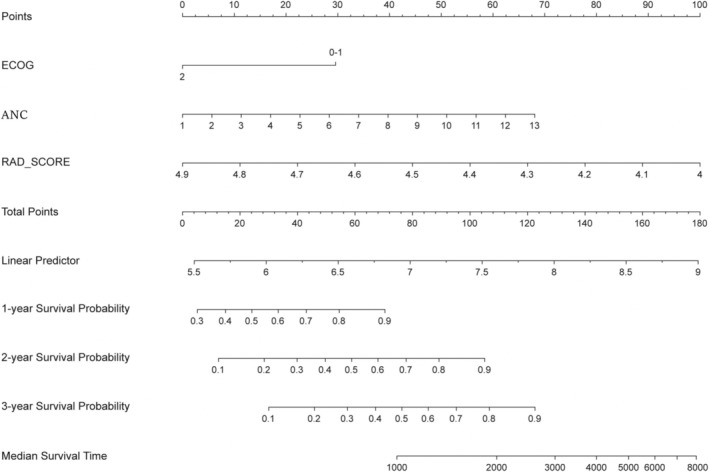
The nomogram combined with the Rad‐score and the independent clinical risk factors, including ECOG and neutrophil, to predict the risk of OS at 1, 2, and 3 years.

In our analysis of the training cohort samples, we performed univariate Cox regression using a combination of clinical variables and Rad‐scores. The outcomes of this analysis are presented in Table 2. Subsequently, in the subsequent multivariate stepwise Cox regression analysis, we included clinical variables that had exhibited statistical significance in the univariate analysis. The findings of this multivariate analysis are detailed in Table 3. Notably, ECOG score, ANC, and Rad‐score emerged as statistically significant independent prognostic factors for predicting OS. Based on these findings, we proceeded to construct a nomogram for personalized OS prediction, as depicted in Figure 4.

**TABLE 2:** Univariate Cox regression on clinical variables of the training cohort samples.

Variables	HR	HR (95%CI)	*p*
Age	1.035	0.998–1.074	0.067
BMI	1.102	0.972–1.248	0.128
Baseline NLR	0.950	0.816–1.106	0.508
ANC	0.754	0.587–0.967	0.026
ALC	0.669	0.406–1.101	0.114
PI‐RADS score			
3	Reference	Reference	
4	1.621	0.361–7.266	0.528
5	1.889	0.555, 6.438	0.309
Clinical T stage			
T2	Reference	Reference	
T3a	0.462	0.057–3.779	0.472
T3b	1.539	0.584–4.056	0.384
T4	1.734	0.708–4.247	0.228
Lymph node involvement			
N0	Reference	Reference	
N+	0.928	0.491–1.792	0.847
Bone metastases			
No	Reference	Reference	
Yes	0.951	0.436–2.074	0.899
ISUP			
≤ 4	Reference	Reference	
5	3.132	1.209–8.109	0.019
Chemotherapy			
No	Reference	Reference	
Yes	0.671	0.334–1.345	0.261
ECOG score			
0–1	Reference	Reference	
2	2.829	1.291–6.200	0.009
PSA as diagnosis			
< 100	Reference	Reference	
≥ 100	0.815	0.437–1.520	0.521
Rad_score	54.778	6.287–477.311	< 0.001

Abbreviations: ALC = absolute lymphocyte count, ANC = absolute neutrophil count, BMI = body mass index, ECOG score = Eastern Cooperative Oncology Group score, ISUP grading group = International Society of Urological Pathology grading group, NLR = neutral–lymphocyte ratio.

**TABLE 3:** Multivariate Cox regression on statistically significant clinical variables in the univariate analysis.

Variables	HR	HR (95%CI)	*p*
ANC	0.740	0.578–0.949	0.018
ISUP grading group			
≤ 4	Reference	Reference	
5	2.581	0.946–7.042	0.064
ECOG score			
0–1	Reference	Reference	
2	4.352	1.880–10.076	< 0.001
Rad_score	52.308	4.822,567.375	0.001

Abbreviations: ANC = absolute neutrophil count, ECOG score = Eastern Cooperative Oncology Group score, ISUP grading group = International Society of Urological Pathology grading group, Rad_score = radiomics sore.

### 3.6 Model Assessment

We compared the performance of the two models, the model without Rad‐score incorporates the independent clinical predictors including ECOG score and ANC. The model with Rad‐score incorporates the independent clinical predictors including ECOG score, ANC, and Rad‐score.

**FIGURE 5:**
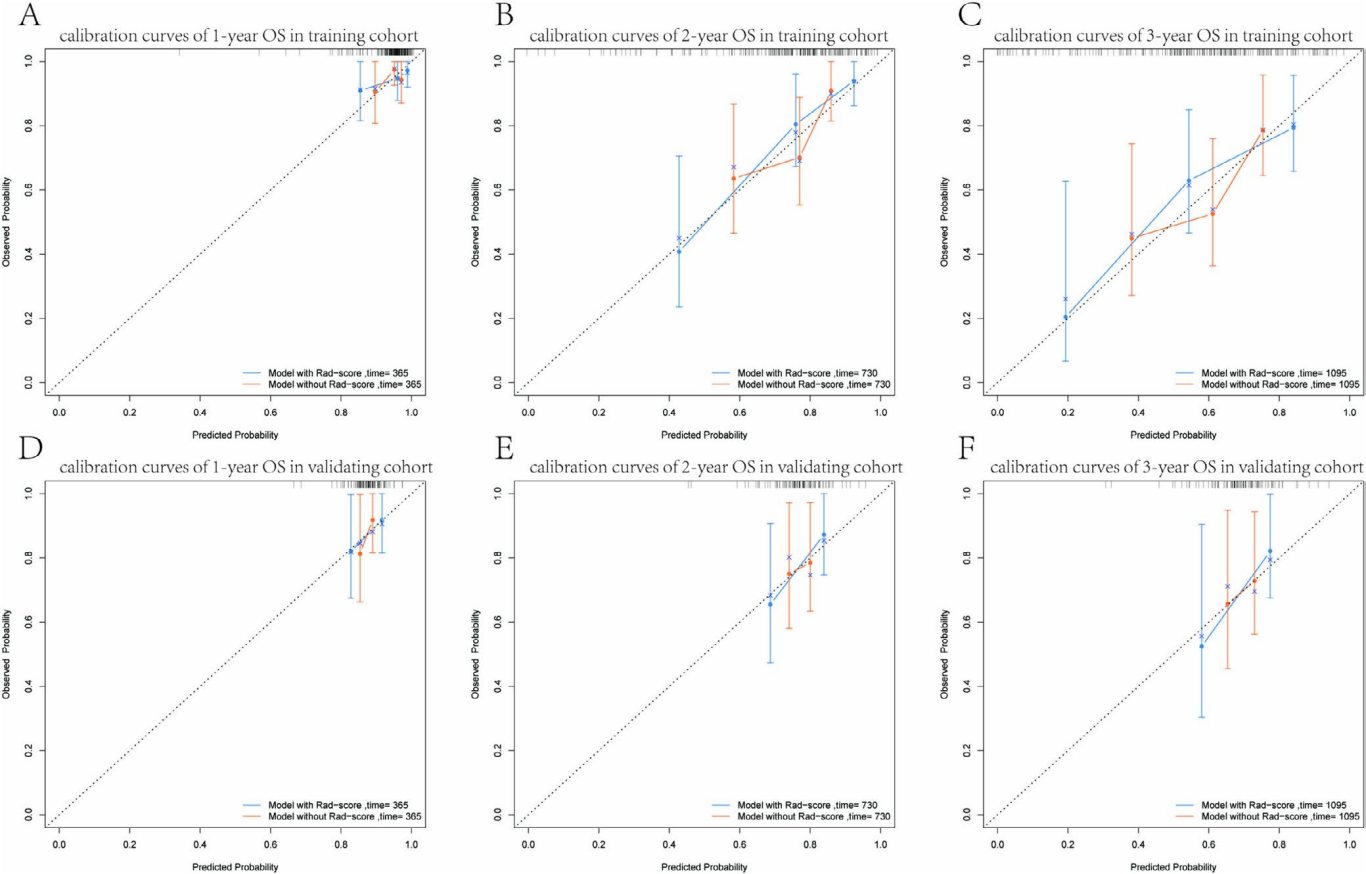
The calibration curves of the models for evaluating the OS in the training and validating cohorts. (A–C) The calibration curves of the comparison between the model with or without Rad‐score to predict the 1‐, 2‐, and 3‐year OS in the training cohort. (D–F) The calibration curves of the comparison between the model with or without Rad‐score to predict the 1‐, 2‐, and 3‐year OS in the validating cohort. The diagonal dotted line represents the ideal state, and the solid red line represents the actual predictive value: The closer it is to the diagonal dotted line, the better the predictive power.

Following the construction of calibration curves, we measured the level of concordance between the actual outcomes of cases and the predictions made by the model without Rad‐score and model with Rad‐score. Figure 5A–C display these calibration curves, which pertain to 1‐, 2‐, and 3‐year OS. This validation process was also extended to the validating group, and the results are illustrated in Figure 5D–F. A model with superior accuracy will exhibit a closer alignment to the diagonal dotted line, which signifies a strong agreement between the model's predictions and the actual clinical observations.

**FIGURE 6:**
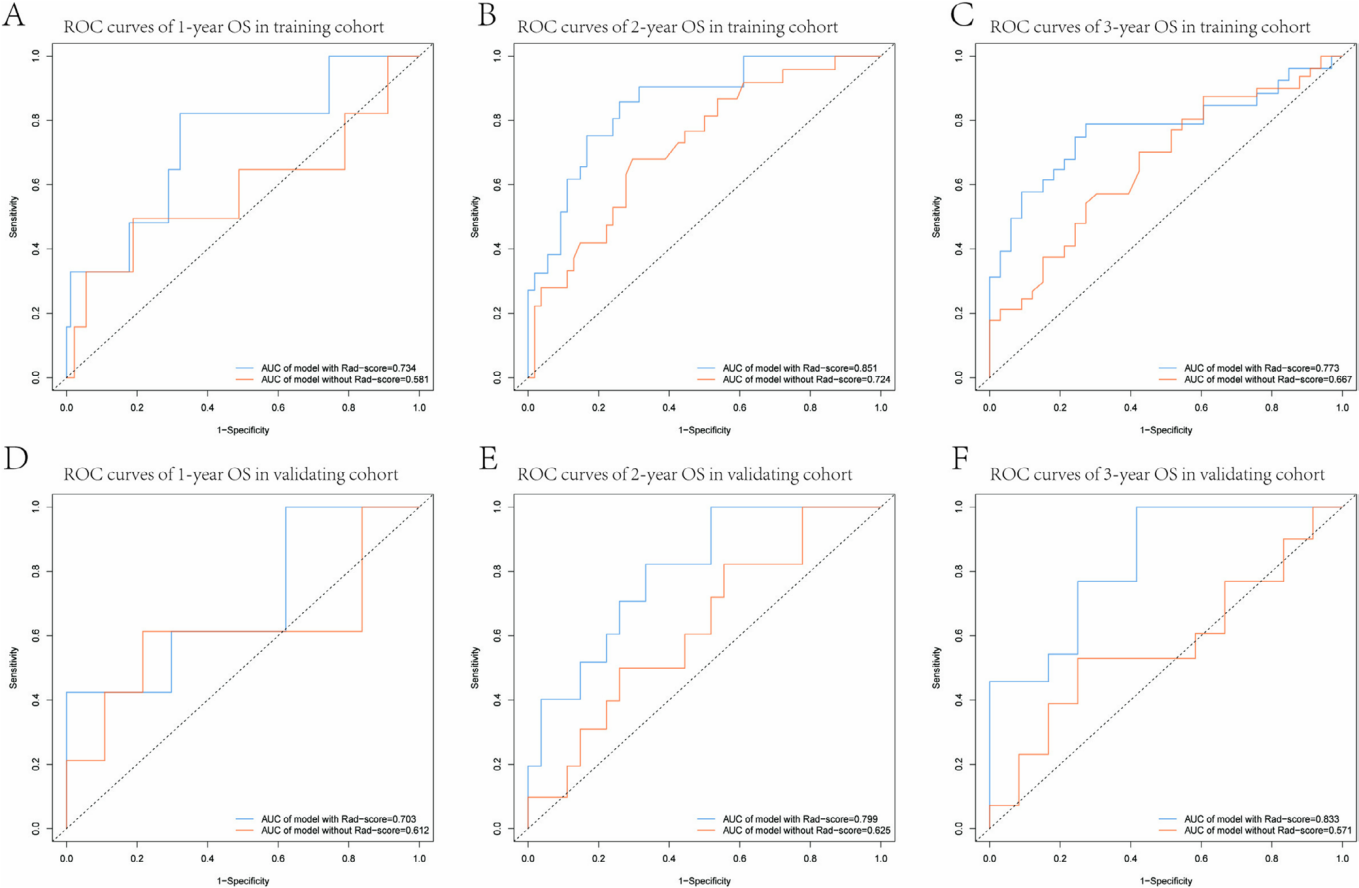
The ROC curves of the models for evaluating the OS in the training and validating cohorts. (A–C) The ROC curves of the comparison between the model with or without Rad‐score to predict the 1‐, 2‐, and 3‐year OS in the training cohort. (D–F) The ROC curves of the comparison between the model with or without Rad‐score to predict the 1‐, 2‐, and 3‐year OS in the validating cohort. It was found that the model with Rad‐score was better than the model without Rad‐score in predicting OS.

We also constructed ROC curves for the two models. Figure 6A–C illustrate these time‐dependent ROC curves for the training cohort across 1‐, 2‐, and 3‐year OS predictions; this validation was also similarly applied to the validating group as shown in Figure 6D–F. Figure S3 displays the time‐dependent AUC curves for both the training cohort and validating cohort. The results clearly indicate that clinically independent prognostic factors, including Rad‐scores, significantly enhanced the predictive accuracy and clinical diagnostic capability of the model. In the training cohort, the AUC values improved notably with the inclusion of Rad‐score parameters: 1‐year OS (AUC 0.734 vs. 0.581), 2‐year OS (AUC 0.851 vs. 0.724), and 3‐year OS (AUC 0.773 vs. 0.667). Similarly, in the validating cohort, the model's performance was also enhanced with Rad‐score parameters: 1‐year OS (AUC 0.703 vs. 0.612), 2‐year OS (AUC 0.799 vs. 0.625), and 3‐year OS (AUC 0.833 vs. 0.571). These results underscore the value of Rad‐scores in improving the model's predictive accuracy and clinical utility.

**FIGURE 7:**
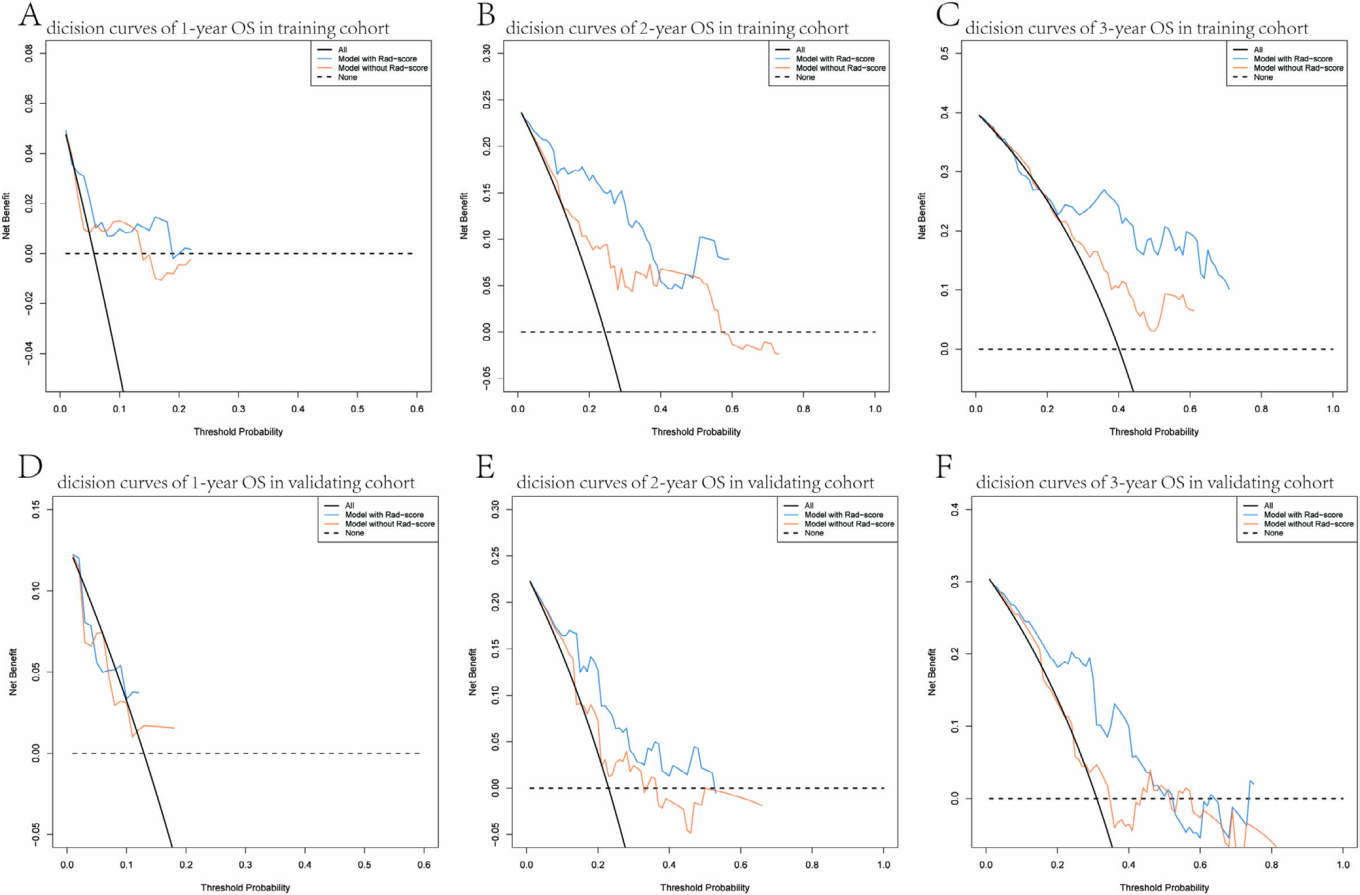
The DCA curves of the models in training and validating cohorts. (A–C) The DCA curves of the comparison between the model with or without Rad‐score to predict the 1‐, 2‐, and 3‐year OS in the training cohort. (D–F) The DCA curves of the comparison between the model with or without Rad‐score to predict the 1‐, 2‐, and 3‐year OS in the validating cohort. DCA curves showed that the model with Rad‐score benefits patients in the prediction of OS at 1, 2, and 3 years.

In both the training and validating cohorts of the clinical model, a Rad‐score was deliberately omitted to illustrate the specific contribution of the Rad‐score. Figure 7 presents decision curves for both the training cohorts (Figure 7A–C) and the validation cohorts (Figure 7D–F) with and without Rad‐scores, respectively. These results clearly indicate that incorporating Rad‐scores improves the clinical prediction performance, underscoring its potential clinical utility.

## 4 Discussion

To summarize, we have created a nomogram that relies on Rad‐score, ECOG score, and ANC analysis for the purpose of individually predicting OS in oligometastatic PCa cases. This nomogram underwent validation, and the OS of the cases was assessed at 1, 2, and 3 years. The findings clearly indicate that such a model combining Rad‐score with clinical factors substantially enhances predictive accuracy when compared to models that solely incorporate clinical factors or Rad‐score in isolation.

The prefix “oligo‐” signifies “few” or “small,” while “metastatic” indicates that cancer has extended from the initial tumor to other areas of the body. Oligometastatic PCa refers to a condition where cancer cells originating in the prostate migrate and develop new tumors in only a limited number of other body regions. However, there is currently no standardized definition of oligometastatic PCa. Several researchers applied stringent inclusion criteria. Two studies set the threshold at bulky positive nodes (< 5 cm) or no more than six bone metastases [35, 36], while others limited the number of metastases to five or fewer [23, 24, 25]. Another article established a threshold of four or fewer [37]. Some studies even set a stricter requirement of three or fewer metastases [13, 26, 38]. Notably, in the study conducted by Tabata and colleagues, their focus was on bony metastases with lesions less than 50% the size of a vertebral body [23]. This approach adhered to the extent of disease criteria previously articulated by Soloway and colleagues [39]. Furthermore, two other studies narrowed down their inclusion criteria to either bone or lymph node metastases [26, 27]. A definition of oligometastatic PCa with up to five detectable lesions was described in a review of definitions of oligometastatic PCa, which has been widely adopted and is commonly used. Singh and colleagues conducted a study to examine the relationship between the number of metastatic lesions in patients and their survival rates. They discovered that men with five or fewer lesions had survival rates similar to those without any metastases and significantly better survival rates compared to those with more than five lesions (*p* = 0.02) [40]. Therefore, in this study, we defined oligometastatic PCa as having only lymph node and bone metastases with no more than five metastases and explored the prognostic factors of oligometastatic PCa.

In this study, we analyzed the clinical T‐staging of all patients who underwent mp‐MRI evaluation. The majority of patients were classified as T3a, T3b, or T4, which is consistent with the inclusion of oligometastatic PCa patients in more advanced stages. Specifically, T4 and higher stages accounted for approximately 48.5% of the cohort, reflecting the typical distribution seen in this patient population. However, it is noteworthy that around 17.6% of the patients were classified as T2, representing an earlier stage of oligometastatic disease. Interestingly, some of the T2 patients displayed imaging features that were more characteristic of higher T stages, suggesting that despite their lower clinical staging, these tumors may exhibit more aggressive biological behavior. This raises the possibility that mp‐MRI may identify subtle but significant differences in tumor biology even in patients with early‐stage disease. Such findings highlight the potential of mp‐MRI in identifying patients with seemingly early‐stage disease who may still be at risk for worse outcomes. This observation warrants further investigation into the imaging characteristics of T2 patients and their correlation with clinical outcomes. Future studies with larger cohorts could help clarify the role of mp‐MRI in different T stages, potentially leading to more tailored treatment strategies. Furthermore, exploring the underlying biological mechanisms that contribute to these imaging findings could provide new insights into the progression and management of oligometastatic PCa.

The field of prostate mp‐MRI has undergone substantial advancements since its inception. Enhanced MRI resolution and the incorporation of functional images have resulted in significantly improved accuracy rates for excluding clinically significant diseases [41]. This has facilitated more precise targeting during biopsy procedures, reducing the likelihood of detecting clinically inconsequential conditions [42]. Globally, the adoption of PIRADS as an evidence‐based consensus has standardized both the acquisition and interpretation of prostate mp‐MRI scans [43]. Several pretreatment MRI findings were significant prognostic factors in patients with PCa who underwent RT. Researchers have confirmed that extraprostatic extension (ECE), seminal vesical invasion (SVI), and larger tumor size based on mp‐MRI were significant prognostic factors of BCR [44, 45, 46]. Two studies found that SVI was a substantial risk factor for the development of metastasis [47, 48]. Prostate mp‐MRI contains a variety of indicators related to the prognosis of PCa patients, which is why we chose to establish a prognostic radiomics prediction model for oligometastatic PCa patients based on prostate mp‐MRI. We used univariate Cox regression to evaluate various clinical variables and Rad‐score with the aim of predicting OS. We found only ECOG scores, ISUP grading group, ANC, and Rad‐score showed statistical significance. ECOG performance status has been consistently used to predict cancer prognosis, with poorer performance status correlating with worse survival outcomes [49, 50, 51]. A meta‐analysis demonstrated that castration‐resistant prostate cancer (CRPC) patients with an ECOG performance status > 1 had a significantly higher mortality risk than those with lower ECOG performance status [52]. The research conducted by Guang‐Xi Sun et al. revealed that patients with lower ISUP grading in the context of CRPC experience extended castration‐resistant PCa‐free survival (CFS) and OS [53]. Interestingly, in our study, higher ANC was associated with better OS, which differs from findings in many other cancer types where elevated ANC is typically a marker of poorer prognosis due to systemic inflammation and tumor burden [54, 55, 56]. Although fewer studies have assessed the inhibitory effects of neutrophils on cancer, very interesting data have been reported. For example, neutrophils slow cancer growth by controlling microbial populations and cancer‐associated inflammation [57]. Neutrophils produce chemokines that recruit T cells and other leukocytes to indirectly kill cancer cells [58]. Neutrophils acquire the characteristics of antigen‐presenting cells (APCs) in the early stage and thus might stimulate the proliferation of T cells to protect against tumor metastasis [59]. This inverse relationship between higher ANC with better prognosis could be attributed to a more robust immune response in patients with higher ANC, indicating a stronger innate immune defense against tumor progression. Additionally, patients with higher ANC may exhibit better tolerance to therapies such as ADT, which could enhance treatment outcomes. Another possibility is that higher ANC reflects less compromised bone marrow function, suggesting that these patients have not experienced significant immunosuppression, which may be critical for improving survival in oligometastatic PCa. The result of multivariate Cox regression shows only ECOG sores, ANC, and Rad‐score present statistically significant as independent predictors. This can be caused by the fact that the Rad‐score contains information on ISUP grading group, and previous studies have been able to confirm this viewpoint [60, 61]. Consequently, we have opted to utilize ECOG scores, ANC, and the Rad‐score as the key factors for constructing and validating a nomogram aimed at predicting OS in patients with oligometastatic PCa.

Currently, there is extensive research on radiomics prediction models that utilize MRI for various aspects of PCa. Francesco Prata et al. have shown that radiomic analysis in patients undergoing targeted fusion biopsies could improve the detection rate of CS PCa and overcome the limitations of the subjective interpretation of MRI images [62]. Ma S et al. developed a model that utilizes a Rad score to predict ECE in prostate patients. This model achieved an impressive AUC of 0.902, surpassing the precision of radiologists [63]. Lin Li et al. created and validated a nomogram, which integrates radiomic and clinicopathologic information to predict postsurgical BCR and adverse pathology in men with PCa [64]. Yushan Jia et al. constructed a hybrid model from radiomics and clinical data showed excellent performance (AUC = 0.926) in predicting PFS in PCa patients and evaluation of treatment response [22]. Hence, the utilization of radiomics offers a noninvasive means to evaluate tumor heterogeneity and has the potential to enhance the development of more effective tumor management strategies. However, there is presently a lack of research regarding the assessment of OS in oligometastatic PCa patients through mp‐MRI radiomics. In this research, we combined radiomic and clinicopathological data to construct a nomogram for forecasting the 1, 2, and 3‐year OS of patients with oligometastatic PCa. The model with Rad‐score demonstrated excellent predictive performance to predict OS at 1, 2, and 3 years, with corresponding AUC values of 0.734, 0.851, and 0.773. The nomogram was validated in the validating cohort; it showed good accuracy with corresponding AUC values of 0.703, 0.799, and 0.833. The model with Rad‐score in this study showed higher accuracy in predicting OS than the model without Rad‐score. In this study, we first developed and validated an MRI‐based radiomics nomogram to predict the prognostic risk of patients with oligometastatic PCa. Our model offers a unique advantage over existing radiomics‐based predictive models by integrating ANC and ECOG performance status alongside radiomic features. By incorporating inflammatory markers like ANC, we enhance the model's predictive power. Our findings suggest that elevated ANC may serve as a positive prognostic factor in specific contexts, likely reflecting individual variations in patient characteristics and treatment responses. This underscores the complex role of systemic inflammation in cancer progression, which has become increasingly recognized. This integrative approach offers a more comprehensive prognostic tool than models based solely on imaging or clinical parameters.

Nonetheless, there are several limitations to our study. Firstly, the sample size is relatively small, and we conducted the research at a single institution, which may limit the generalizability of our findings. Additionally, the retrospective nature of the study could introduce selection bias. Future investigations should encompass multiple centers and employ prospective study designs to further validate the effectiveness and applicability of our combined prediction model across diverse patient populations. Furthermore, our radiomics analysis focused exclusively on T2WI and ADC images, excluding dynamic enhancement images from consideration. Integrating multiparametric data analysis may have the potential to enhance the overall quality and performance of the model. Additionally, the process of extracting and selecting radiomics features may be influenced by interobserver variability, although we took steps to minimize this by involving experienced radiologists for manual ROI segmentation. Lastly, it is crucial to externally validate our prediction model to evaluate its performance in various cohorts and clinical settings, thereby increasing its practical utility in the field.

## 5 Conclusion

In this study, we have developed a radiomics nomogram that combines ECOG scores and ANC with a Rad‐score derived from mp‐MRI images. Our findings demonstrate that the predictive capability of this model, which incorporates radiomics features, surpasses that of clinical indicators. This enhanced model could serve as a valuable tool for more accurately evaluating the prognostic risk in patients with de novo oligometastatic PCa, offering clinicians valuable guidance for optimizing treatment strategies and improving patient outcomes.

## Author Contributions

**Wen‐Qi Liu:** conceptualization (equal), methodology (equal), writing – original draft (equal). **Yu‐Ting Xue:** data curation (equal), investigation (equal), methodology (equal), writing – original draft (equal). **Xu‐Yun Huang:** formal analysis (equal), writing – original draft (equal). **Bin Lin:** data curation (equal), writing – original draft (equal). **Xiao‐Dong Li:** investigation (equal). **Zhi‐Bin Ke:** investigation (equal). **Dong‐Ning Chen:** formal analysis (equal). **Jia‐Yin Chen:** formal analysis (equal). **Yong Wei:** supervision (equal). **Qing‐Shui Zheng:** supervision (equal). **Xue‐Yi Xue:** project administration (equal), writing – review and editing (equal). **Ning Xu:** conceptualization (equal), project administration (equal), writing – review and editing (equal).

## Ethics Statement

This retrospective study was conducted in accordance with the Declaration of Helsinki (as revised in 2013) and was approved by the Ethics Committee of the First Affiliated Hospital of Fujian Medical University (Approved No. of Ethic Committee: MTCA and ECFAH of FMU [2024]646). The requirement of informed consent from the study participants was waived due to the retrospective nature of this study.

## Consent

The authors have nothing to report.

## Conflicts of Interest

The authors declare no conflicts of interest.

## Supporting information


**Figure S1.** ANC analysis of total patients with oligometastatic PCa. ANC level chart (A). The Kaplan–Meier survival analysis demonstrates the difference in overall survival (OS) between high and low ANC groups (B).

**Figure S2.** Utilize LASSO regression for radiomics feature selection. Employ 10‐fold cross‐validation to tune the parameters lambda for PCa (A) features. Referencing the coefficient curve plot generated by the optimal log(lambda) sequence, the coefficients PCa (B) features comprise four values.

**Figure S3.** Time‐dependent area under the curves (AUCs) of the model with Rad‐score and model without the Rad‐score in the training cohort (A) and validating (B) cohort.

Table S1.

## Data Availability

The data are not publicly available due to the containing information that could compromise the privacy of research participants.
