# Supplementary figures and images for: Development and Validation of an MRI‐Based Radiomics Nomogram to Predict the Prognosis of De Novo Oligometastatic Prostate Cancer Patients
Source: Cancer Med. 2024 Dec 20;13(24):e70481. doi: 10.1002/cam4.70481 (PMC11660381; doi:10.1002/cam4.70481)

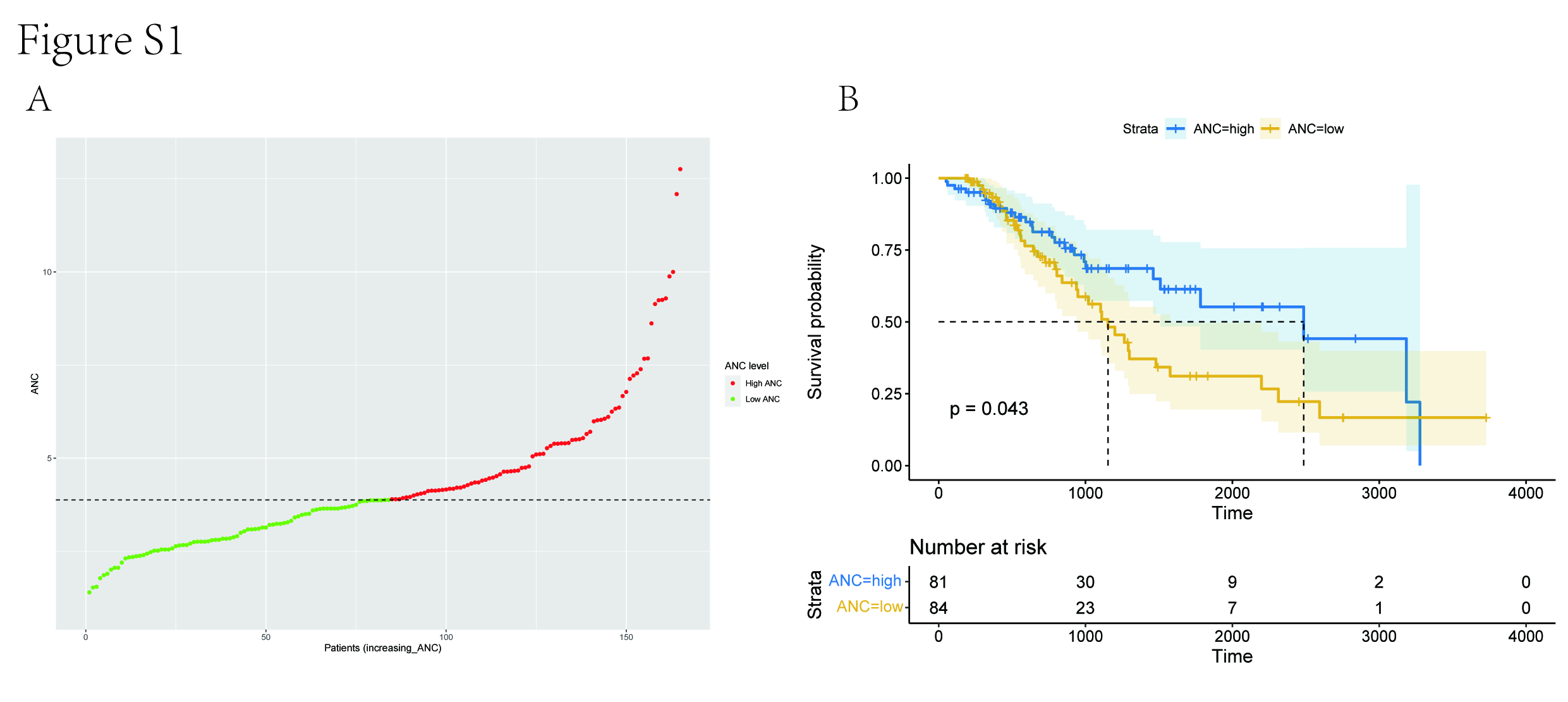

Supplement: Supplementary file 1 — Figure S1. ANC analysis of total patients with oligometastatic PCa. ANC level chart (A). The Kaplan–Meier survival analysis demonstrates the difference in overall survival (OS) between high and low ANC groups (B). [file CAM4-13-e70481-s003.tif]

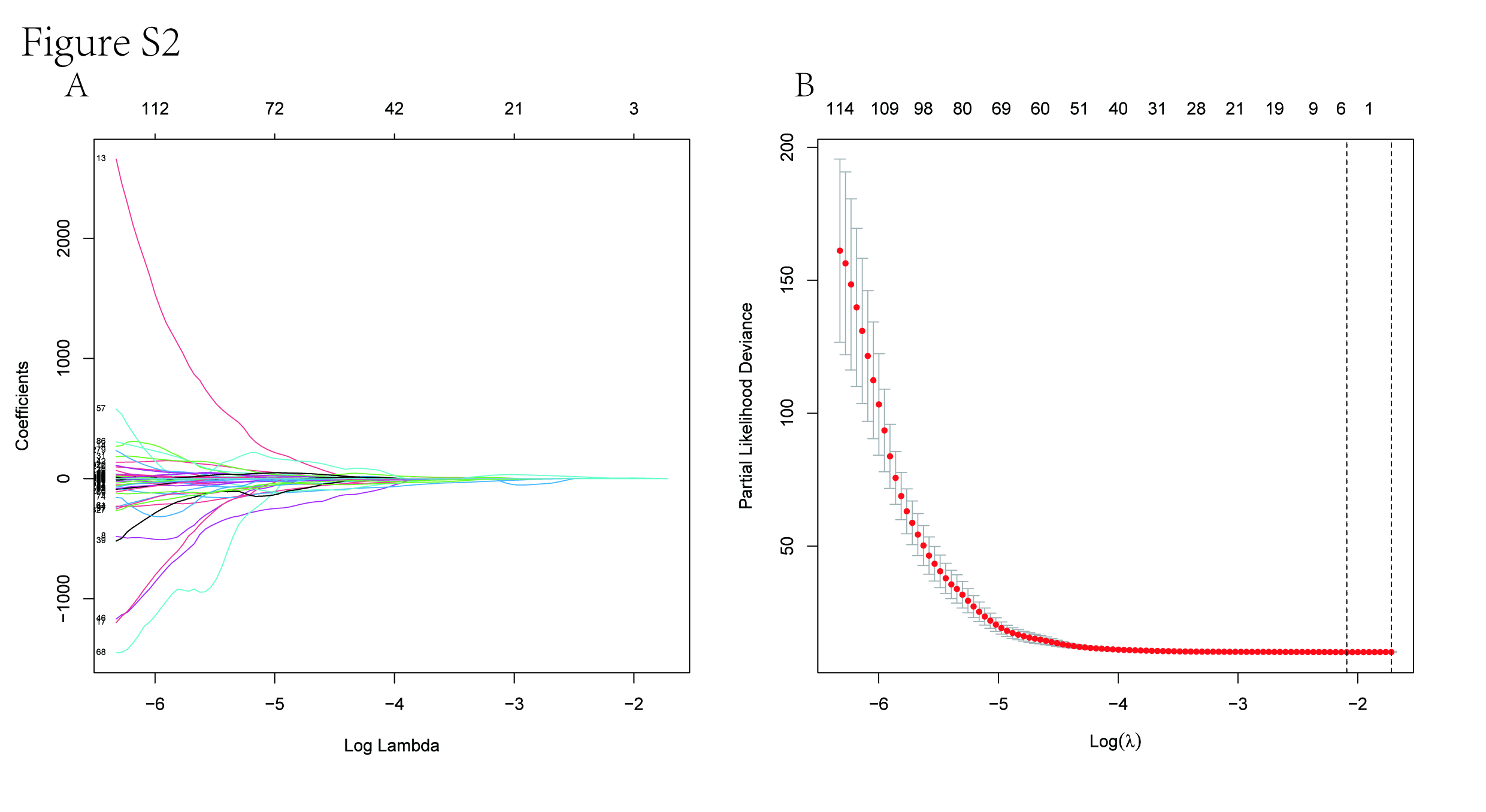

Supplement: Supplementary file 2 — Figure S2. Utilize LASSO regression for radiomics feature selection. Employ 10‐fold cross‐validation to tune the parameters lambda for PCa (A) features. Referencing the coefficient curve plot generated by the optimal log(lambda) sequence, the coefficients PCa (B) features comprise four values. [file CAM4-13-e70481-s004.tif]

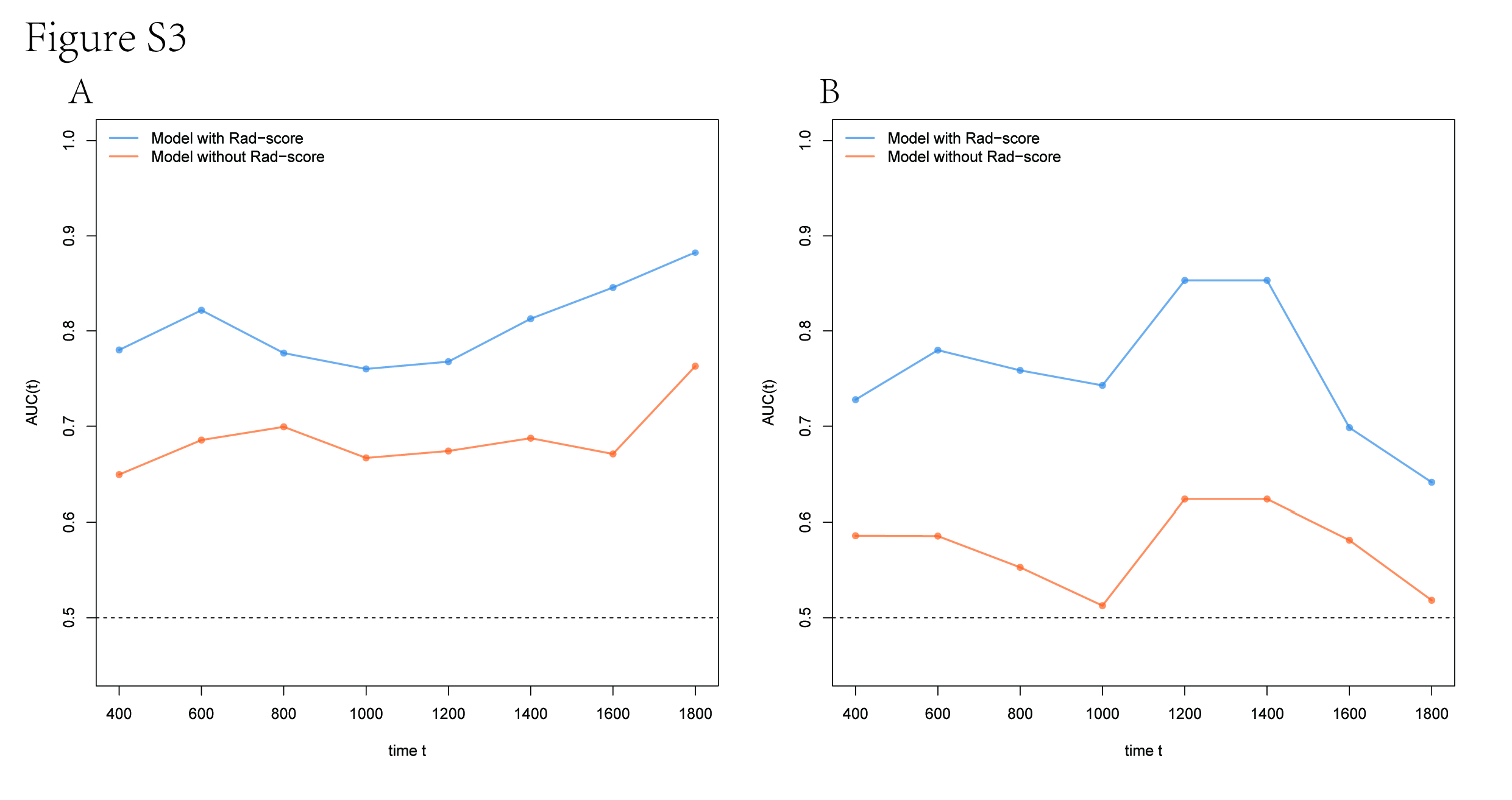

Supplement: Supplementary file 3 — Figure S3. Time‐dependent area under the curves (AUCs) of the model with Rad‐score and model without the Rad‐score in the training cohort (A) and validating (B) cohort. [file CAM4-13-e70481-s001.tif]
