# Supplementary material for: Development and Validation of an MRI‐Based Radiomics Nomogram to Predict the Prognosis of De Novo Oligometastatic Prostate Cancer Patients
Source: Cancer Med. 2024 Dec 20;13(24):e70481. doi: 10.1002/cam4.70481 (PMC11660381; doi:10.1002/cam4.70481)
Supplement: Supplementary file 4 — Table S1. [file CAM4-13-e70481-s002.docx]

**Table S1**

| Variable | ***P*** Value |
| --- | --- |
| diagnostics_Image-original_Mean_T2WI | 0.00354552 |
| diagnostics_Mask-original_VolumeNum_T2WI | 0.00336297 |
| diagnostics_Mask-interpolated_VolumeNum_T2WI | 0.01620714 |
| original_shape_Maximum2DDiameterSlice_T2WI | 0.03704127 |
| original_shape_Sphericity_T2WI | 0.0053923 |
| original_firstorder_Kurtosis_T2WI | 0.00745659 |
| original_firstorder_Skewness_T2WI | 0.00495904 |
| original_glcm_Idmn_T2WI | 0.04716779 |
| original_glcm_Idn_T2WI | 0.01873102 |
| original_gldm_LargeDependenceHighGrayLevelEmphasis_T2WI | 0.02644411 |
| exponential_firstorder_InterquartileRange_T2WI | 0.03383771 |
| exponential_firstorder_RobustMeanAbsoluteDeviation_T2WI | 0.02504968 |
| exponential_glcm_Idmn_T2WI | 0.041190539 |
| exponential_glcm_Idn_T2WI | 0.01812615 |
| exponential_gldm_SmallDependenceLowGrayLevelEmphasis_T2WI | 0.01537342 |
| exponential_ngtdm_Contrast_T2WI | 0.04238005 |
| gradient_firstorder_Kurtosis_T2WI | 0.03220973 |
| gradient_firstorder_Skewness_T2WI | 0.00890003 |
| gradient_glcm_Correlation_T2WI | 0.00317736 |
| gradient_glcm_Idmn_T2WI | 0.03948574 |
| gradient_glcm_Idn_T2WI | 0.01451739 |
| gradient_glcm_Imc1_T2WI | 0.00909608 |
| gradient_glcm_Imc2_T2WI | 0.0093832 |
| gradient_glcm_MCC_T2WI | 0.0065229 |
| gradient_glrlm_RunEntropy_T2WI | 0.04649279 |
| gradient_glszm_LowGrayLevelZoneEmphasis_T2WI | 0.01795288 |
| lbp-2D_glszm_GrayLevelNonUniformity_T2WI | 0.01620714 |
| lbp-2D_glszm_ZoneVariance_T2WI | 0.00462093 |
| logarithm_glcm_Autocorrelation_T2WI | 0.02512139 |
| logarithm_glcm_Imc2_T2WI | 0.04071747 |
| logarithm_gldm_DependenceEntropy_T2WI | 0.03349937 |
| logarithm_gldm_HighGrayLevelEmphasis_T2WI | 0.02499689 |
| logarithm_gldm_LargeDependenceHighGrayLevelEmphasis_T2WI | 0.00891324 |
| logarithm_glrlm_HighGrayLevelRunEmphasis_T2WI | 0.02461095 |
| logarithm_glrlm_LongRunHighGrayLevelEmphasis_T2WI | 0.0123494 |
| logarithm_glrlm_ShortRunHighGrayLevelEmphasis_T2WI | 0.02940719 |
| logarithm_glszm_HighGrayLevelZoneEmphasis_T2WI | 0.01894163 |
| logarithm_glszm_SmallAreaHighGrayLevelEmphasis_T2WI | 0.0274645 |
| logarithm_glszm_ZoneEntropy_T2WI | 0.03258433 |
| square_firstorder_Skewness_T2WI | 0.03003511 |
| square_glcm_Idmn_T2WI | 0.03516192 |
| square_glcm_Idn_T2WI | 0.01327441 |
| square_glcm_MaximumProbability_T2WI | 0.02520178 |
| square_gldm_DependenceNonUniformityNormalized_T2WI | 0.01101273 |
| square_gldm_DependenceVariance_T2WI | 0.02238126 |
| square_gldm_SmallDependenceLowGrayLevelEmphasis_T2WI | 0.01627968 |
| square_glrlm_RunLengthNonUniformityNormalized_T2WI | 0.04824171 |
| square_ngtdm_Contrast_T2WI | 0.03826089 |
| squareroot_firstorder_Kurtosis_T2WI | 0.02462457 |
| squareroot_firstorder_Range_T2WI | 0.03910735 |
| squareroot_firstorder_Skewness_T2WI | 0.01716326 |
| squareroot_glcm_Idmn_T2WI | 0.04261579 |
| squareroot_glcm_Idn_T2WI | 0.01976082 |
| squareroot_gldm_LargeDependenceHighGrayLevelEmphasis_T2WI | 0.01170238 |
| squareroot_glrlm_HighGrayLevelRunEmphasis_T2WI | 0.02782194 |
| squareroot_glrlm_LongRunHighGrayLevelEmphasis_T2WI | 0.01683517 |
| squareroot_glszm_HighGrayLevelZoneEmphasis_T2WI | 0.01599438 |
| squareroot_glszm_SmallAreaHighGrayLevelEmphasis_T2WI | 0.02131135 |
| wavelet-LLH_firstorder_Minimum_T2WI | 0.02178056 |
| wavelet-LLH_glcm_Idmn_T2WI | 0.03053157 |
| wavelet-LLH_glcm_Idn_T2WI | 0.02114346 |
| wavelet-LLH_glcm_JointAverage_T2WI | 0.01979233 |
| wavelet-LLH_glcm_SumAverage_T2WI | 0.01979232 |
| wavelet-LLH_gldm_DependenceEntropy_T2WI | 0.0290803 |
| wavelet-LLH_gldm_DependenceNonUniformityNormalized_T2WI | 0.01675969 |
| wavelet-LLH_gldm_LargeDependenceLowGrayLevelEmphasis_T2WI | 0.03165877 |
| wavelet-LLH_gldm_LowGrayLevelEmphasis_T2WI | 0.01890907 |
| wavelet-LLH_gldm_SmallDependenceLowGrayLevelEmphasis_T2WI | 0.03981181 |
| wavelet-LLH_glrlm_LongRunLowGrayLevelEmphasis_T2WI | 0.04668159 |
| wavelet-LLH_glrlm_LowGrayLevelRunEmphasis_T2WI | 0.01822872 |
| wavelet-LLH_glrlm_ShortRunLowGrayLevelEmphasis_T2WI | 0.01654497 |
| wavelet-LLH_glszm_GrayLevelNonUniformityNormalized_T2WI | 0.0029192 |
| wavelet-LLH_glszm_LowGrayLevelZoneEmphasis_T2WI | 0.02306774 |
| wavelet-LLH_glszm_SmallAreaEmphasis_T2WI | 0.0276967 |
| wavelet-LLH_glszm_SmallAreaLowGrayLevelEmphasis_T2WI | 0.03219981 |
| wavelet-LLH_glszm_ZoneEntropy_T2WI | 0.01152311 |
| wavelet-LLH_ngtdm_Contrast_T2WI | 0.01589691 |
| wavelet-LHL_firstorder_Kurtosis_T2WI | 8.9078E-06 |
| wavelet-LHL_firstorder_Maximum_T2WI | 0.00400149 |
| wavelet-LHL_firstorder_Minimum_T2WI | 0.00173238 |
| wavelet-LHL_firstorder_Range_T2WI | 0.00180762 |
| wavelet-LHL_firstorder_Skewness_T2WI | 0.01800701 |
| wavelet-LHL_glcm_Autocorrelation_T2WI | 0.00350274 |
| wavelet-LHL_glcm_ClusterShade_T2WI | 0.00565276 |
| wavelet-LHL_glcm_Idmn_T2WI | 0.00316841 |
| wavelet-LHL_glcm_Idn_T2WI | 0.00037665 |
| wavelet-LHL_glcm_JointAverage_T2WI | 0.00182509 |
| wavelet-LHL_glcm_MCC_T2WI | 0.00043134 |
| wavelet-LHL_glcm_SumAverage_T2WI | 0.00182508 |
| wavelet-LHL_gldm_HighGrayLevelEmphasis_T2WI | 0.00366319 |
| wavelet-LHL_gldm_LargeDependenceHighGrayLevelEmphasis_T2WI | 0.00106068 |
| wavelet-LHL_gldm_LowGrayLevelEmphasis_T2WI | 0.04114065 |
| wavelet-LHL_gldm_SmallDependenceHighGrayLevelEmphasis_T2WI | 0.02398221 |
| wavelet-LHL_gldm_SmallDependenceLowGrayLevelEmphasis_T2WI | 0.03349312 |
| wavelet-LHL_glrlm_HighGrayLevelRunEmphasis_T2WI | 0.00367257 |
| wavelet-LHL_glrlm_LongRunHighGrayLevelEmphasis_T2WI | 0.00201795 |
| wavelet-LHL_glrlm_LongRunLowGrayLevelEmphasis_T2WI | 0.04199592 |
| wavelet-LHL_glrlm_LowGrayLevelRunEmphasis_T2WI | 0.04393714 |
| wavelet-LHL_glrlm_ShortRunHighGrayLevelEmphasis_T2WI | 0.00449485 |
| wavelet-LHL_glrlm_ShortRunLowGrayLevelEmphasis_T2WI | 0.04154226 |
| wavelet-LHL_glszm_GrayLevelVariance_T2WI | 0.0322708 |
| wavelet-LHL_glszm_HighGrayLevelZoneEmphasis_T2WI | 0.0039825 |
| wavelet-LHL_glszm_SmallAreaHighGrayLevelEmphasis_T2WI | 0.00636778 |
| wavelet-LHL_ngtdm_Complexity_T2WI | 0.01346043 |
| wavelet-LHL_ngtdm_Contrast_T2WI | 0.00456641 |
| wavelet-LHH_firstorder_Kurtosis_T2WI | 0.00012057 |
| wavelet-LHH_firstorder_Maximum_T2WI | 0.00681054 |
| wavelet-LHH_firstorder_Minimum_T2WI | 0.02485866 |
| wavelet-LHH_firstorder_Range_T2WI | 0.01169624 |
| wavelet-LHH_glcm_Idmn_T2WI | 0.0441466 |
| wavelet-LHH_glcm_Idn_T2WI | 0.01702444 |
| wavelet-LHH_glcm_JointAverage_T2WI | 0.03491204 |
| wavelet-LHH_glcm_SumAverage_T2WI | 0.034912 |
| wavelet-LHH_gldm_LargeDependenceHighGrayLevelEmphasis_T2WI | 0.02527491 |
| wavelet-LHH_gldm_LargeDependenceLowGrayLevelEmphasis_T2WI | 0.03727487 |
| wavelet-LHH_gldm_LowGrayLevelEmphasis_T2WI | 0.03434077 |
| wavelet-LHH_gldm_SmallDependenceLowGrayLevelEmphasis_T2WI | 0.03633197 |
| wavelet-LHH_glrlm_LongRunHighGrayLevelEmphasis_T2WI | 0.01775286 |
| wavelet-LHH_glrlm_LongRunLowGrayLevelEmphasis_T2WI | 0.03257532 |
| wavelet-LHH_glrlm_LowGrayLevelRunEmphasis_T2WI | 0.0323109 |
| wavelet-LHH_glszm_GrayLevelVariance_T2WI | 0.01876342 |
| wavelet-LHH_glszm_ZoneEntropy_T2WI | 0.01745813 |
| wavelet-LHH_ngtdm_Complexity_T2WI | 0.01704938 |
| wavelet-LHH_ngtdm_Contrast_T2WI | 0.04210155 |
| wavelet-HLL_firstorder_Kurtosis_T2WI | 0.01605377 |
| wavelet-HLL_glcm_Idmn_T2WI | 0.01348718 |
| wavelet-HLL_glcm_Idn_T2WI | 0.02246559 |
| wavelet-HLL_glcm_Imc2_T2WI | 0.01364481 |
| wavelet-HLL_glcm_InverseVariance_T2WI | 0.04534472 |
| wavelet-HLL_gldm_DependenceNonUniformityNormalized_T2WI | 0.03242041 |
| wavelet-HLL_ngtdm_Contrast_T2WI | 0.02254632 |
| wavelet-HLH_firstorder_Kurtosis_T2WI | 0.00319004 |
| wavelet-HLH_glcm_Idmn_T2WI | 0.02776096 |
| wavelet-HLH_glcm_Idn_T2WI | 0.01296149 |
| wavelet-HLH_glcm_Imc1_T2WI | 0.00093717 |
| wavelet-HLH_glcm_Imc2_T2WI | 0.03756917 |
| wavelet-HLH_gldm_LargeDependenceLowGrayLevelEmphasis_T2WI | 0.0487996 |
| wavelet-HLH_gldm_LowGrayLevelEmphasis_T2WI | 0.04540694 |
| wavelet-HLH_gldm_SmallDependenceLowGrayLevelEmphasis_T2WI | 0.02770644 |
| wavelet-HLH_glrlm_LongRunLowGrayLevelEmphasis_T2WI | 0.01355362 |
| wavelet-HLH_glrlm_LowGrayLevelRunEmphasis_T2WI | 0.01345482 |
| wavelet-HLH_glrlm_ShortRunLowGrayLevelEmphasis_T2WI | 0.01175961 |
| wavelet-HLH_glszm_GrayLevelVariance_T2WI | 0.03848285 |
| wavelet-HLH_glszm_ZoneEntropy_T2WI | 0.01098428 |
| wavelet-HLH_ngtdm_Contrast_T2WI | 0.04797918 |
| wavelet-HHL_firstorder_Kurtosis_T2WI | 0.00084061 |
| wavelet-HHL_firstorder_Minimum_T2WI | 0.00956275 |
| wavelet-HHL_firstorder_Range_T2WI | 0.02082418 |
| wavelet-HHL_glcm_Autocorrelation_T2WI | 0.00752699 |
| wavelet-HHL_glcm_Idmn_T2WI | 0.00224712 |
| wavelet-HHL_glcm_Idn_T2WI | 0.00048041 |
| wavelet-HHL_glcm_JointAverage_T2WI | 0.01045663 |
| wavelet-HHL_glcm_SumAverage_T2WI | 0.01045662 |
| wavelet-HHL_gldm_DependenceNonUniformityNormalized_T2WI | 0.01575887 |
| wavelet-HHL_gldm_HighGrayLevelEmphasis_T2WI | 0.00849576 |
| wavelet-HHL_gldm_LargeDependenceHighGrayLevelEmphasis_T2WI | 0.00021757 |
| wavelet-HHL_gldm_SmallDependenceLowGrayLevelEmphasis_T2WI | 0.01110498 |
| wavelet-HHL_glrlm_HighGrayLevelRunEmphasis_T2WI | 0.00851371 |
| wavelet-HHL_glrlm_LongRunHighGrayLevelEmphasis_T2WI | 0.00487193 |
| wavelet-HHL_glrlm_ShortRunHighGrayLevelEmphasis_T2WI | 0.01318371 |
| wavelet-HHL_glszm_HighGrayLevelZoneEmphasis_T2WI | 0.00870084 |
| wavelet-HHL_glszm_SmallAreaHighGrayLevelEmphasis_T2WI | 0.01892206 |
| wavelet-HHL_ngtdm_Contrast_T2WI | 0.00144285 |
| wavelet-HHH_firstorder_Kurtosis_T2WI | 0.00014717 |
| wavelet-HHH_firstorder_Maximum_T2WI | 0.03777476 |
| wavelet-HHH_firstorder_Minimum_T2WI | 0.02170834 |
| wavelet-HHH_firstorder_Range_T2WI | 0.02733127 |
| wavelet-HHH_glcm_Autocorrelation_T2WI | 0.01757776 |
| wavelet-HHH_glcm_Correlation_T2WI | 0.01450307 |
| wavelet-HHH_glcm_JointAverage_T2WI | 0.03724 |
| wavelet-HHH_glcm_SumAverage_T2WI | 0.03724008 |
| wavelet-HHH_gldm_HighGrayLevelEmphasis_T2WI | 0.01797654 |
| wavelet-HHH_gldm_LargeDependenceHighGrayLevelEmphasis_T2WI | 0.01134942 |
| wavelet-HHH_gldm_SmallDependenceLowGrayLevelEmphasis_T2WI | 0.02191981 |
| wavelet-HHH_glrlm_HighGrayLevelRunEmphasis_T2WI | 0.0182796 |
| wavelet-HHH_glrlm_LongRunHighGrayLevelEmphasis_T2WI | 0.01137955 |
| wavelet-HHH_glrlm_ShortRunHighGrayLevelEmphasis_T2WI | 0.02543628 |
| wavelet-HHH_glszm_GrayLevelVariance_T2WI | 0.03845117 |
| wavelet-HHH_glszm_HighGrayLevelZoneEmphasis_T2WI | 0.01156394 |
| wavelet-HHH_glszm_SmallAreaHighGrayLevelEmphasis_T2WI | 0.01213416 |
| wavelet-HHH_ngtdm_Complexity_T2WI | 0.03067128 |
| wavelet-LLL_firstorder_Range_T2WI | 0.04152596 |
| wavelet-LLL_firstorder_Skewness_T2WI | 0.01165993 |
| wavelet-LLL_glcm_Idmn_T2WI | 0.04947701 |
| wavelet-LLL_glcm_Idn_T2WI | 0.01794966 |
| wavelet-LLL_gldm_LargeDependenceHighGrayLevelEmphasis_T2WI | 0.02385027 |
| diagnostics_Image-original_Maximum_ADC | 0.00857088 |
| diagnostics_Mask-interpolated_Mean_ADC | 0.00408725 |
| original_shape_MajorAxisLength_ADC | 0.02868795 |
| original_shape_Maximum2DDiameterSlice_ADC | 0.02163526 |
| original_shape_Maximum3DDiameter_ADC | 0.0226969 |
| original_shape_Sphericity_ADC | 0.0008855 |
| original_firstorder_10Percentile_ADC | 0.02302585 |
| original_firstorder_90Percentile_ADC | 0.00598779 |
| original_firstorder_InterquartileRange_ADC | 0.03309726 |
| original_firstorder_Mean_ADC | 0.00408725 |
| original_firstorder_Median_ADC | 0.0035301 |
| original_firstorder_RobustMeanAbsoluteDeviation_ADC | 0.03680008 |
| original_firstorder_RootMeanSquared_ADC | 0.00420021 |
| original_firstorder_Variance_ADC | 0.02332108 |
| original_glcm_ClusterTendency_ADC | 0.01320707 |
| original_glcm_Contrast_ADC | 0.03105671 |
| original_glcm_DifferenceAverage_ADC | 0.01832779 |
| original_glcm_InverseVariance_ADC | 0.03743209 |
| original_glcm_SumSquares_ADC | 0.01851803 |
| original_gldm_DependenceNonUniformityNormalized_ADC | 0.01779709 |
| original_gldm_GrayLevelVariance_ADC | 0.02335159 |
| original_gldm_SmallDependenceEmphasis_ADC | 0.01375641 |
| original_glszm_ZonePercentage_ADC | 0.01516041 |
| original_ngtdm_Contrast_ADC | 0.02935583 |
| original_ngtdm_Strength_ADC | 0.02091516 |
| exponential_glcm_ClusterProminence_ADC | 0.0443955 |
| exponential_glcm_ClusterShade_ADC | 0.04156462 |
| exponential_glcm_Contrast_ADC | 0.04148712 |
| exponential_glcm_DifferenceVariance_ADC | 0.04181715 |
| exponential_gldm_SmallDependenceHighGrayLevelEmphasis_ADC | 0.04679209 |
| exponential_ngtdm_Contrast_ADC | 0.04476665 |
| gradient_firstorder_10Percentile_ADC | 0.01684801 |
| gradient_firstorder_Mean_ADC | 0.02407777 |
| gradient_firstorder_Median_ADC | 0.01032692 |
| gradient_firstorder_RootMeanSquared_ADC | 0.04615447 |
| gradient_glcm_Autocorrelation_ADC | 0.02904887 |
| gradient_glcm_Contrast_ADC | 0.01954338 |
| gradient_glcm_DifferenceAverage_ADC | 0.04806817 |
| gradient_glcm_InverseVariance_ADC | 0.01402993 |
| gradient_glcm_JointAverage_ADC | 0.01944842 |
| gradient_glcm_SumAverage_ADC | 0.01944838 |
| gradient_gldm_DependenceNonUniformityNormalized_ADC | 0.03464138 |
| gradient_gldm_HighGrayLevelEmphasis_ADC | 0.03680587 |
| gradient_gldm_SmallDependenceEmphasis_ADC | 0.01015728 |
| gradient_glrlm_HighGrayLevelRunEmphasis_ADC | 0.046899 |
| gradient_glrlm_LongRunHighGrayLevelEmphasis_ADC | 0.0367759 |
| gradient_glrlm_ShortRunHighGrayLevelEmphasis_ADC | 0.04927656 |
| gradient_glszm_ZonePercentage_ADC | 0.03437213 |
| gradient_ngtdm_Strength_ADC | 0.01417287 |
| lbp-2D_glrlm_RunLengthNonUniformity_ADC | 0.00684735 |
| square_firstorder_10Percentile_ADC | 0.02300714 |
| square_firstorder_90Percentile_ADC | 0.01804967 |
| square_firstorder_Entropy_ADC | 0.04883144 |
| square_firstorder_InterquartileRange_ADC | 0.03497264 |
| square_firstorder_MeanAbsoluteDeviation_ADC | 0.01890347 |
| square_firstorder_Mean_ADC | 0.01977946 |
| square_firstorder_Median_ADC | 0.02630188 |
| square_firstorder_RobustMeanAbsoluteDeviation_ADC | 0.03385952 |
| square_firstorder_RootMeanSquared_ADC | 0.02274229 |
| square_firstorder_Variance_ADC | 0.02644518 |
| square_glcm_Autocorrelation_ADC | 0.02730201 |
| square_glcm_ClusterTendency_ADC | 0.02269199 |
| square_glcm_Contrast_ADC | 0.02045406 |
| square_glcm_DifferenceAverage_ADC | 0.01380161 |
| square_glcm_DifferenceEntropy_ADC | 0.02251963 |
| square_glcm_DifferenceVariance_ADC | 0.03879136 |
| square_glcm_Id_ADC | 0.02939424 |
| square_glcm_Idm_ADC | 0.03230159 |
| square_glcm_InverseVariance_ADC | 0.01643744 |
| square_glcm_JointAverage_ADC | 0.02409798 |
| square_glcm_JointEntropy_ADC | 0.03903398 |
| square_glcm_SumAverage_ADC | 0.024098 |
| square_glcm_SumEntropy_ADC | 0.01111896 |
| square_glcm_SumSquares_ADC | 0.01518081 |
| square_gldm_DependenceNonUniformityNormalized_ADC | 0.02783697 |
| square_gldm_GrayLevelVariance_ADC | 0.02630407 |
| square_gldm_HighGrayLevelEmphasis_ADC | 0.02730597 |
| square_gldm_SmallDependenceEmphasis_ADC | 0.01860275 |
| square_gldm_SmallDependenceHighGrayLevelEmphasis_ADC | 0.03234268 |
| square_glrlm_HighGrayLevelRunEmphasis_ADC | 0.0316822 |
| square_glrlm_LongRunHighGrayLevelEmphasis_ADC | 0.03214945 |
| square_glrlm_ShortRunHighGrayLevelEmphasis_ADC | 0.03188706 |
| square_glszm_ZonePercentage_ADC | 0.01990668 |
| square_ngtdm_Strength_ADC | 0.01268043 |
| squareroot_firstorder_10Percentile_ADC | 0.03589434 |
| squareroot_firstorder_90Percentile_ADC | 0.03358349 |
| squareroot_firstorder_Mean_ADC | 0.0212441 |
| squareroot_firstorder_Median_ADC | 0.01374401 |
| squareroot_firstorder_RootMeanSquared_ADC | 0.02193216 |
| squareroot_ngtdm_Contrast_ADC | 0.0311277 |
| squareroot_ngtdm_Strength_ADC | 0.01489032 |
| wavelet-LLH_gldm_LargeDependenceLowGrayLevelEmphasis_ADC | 0.04618371 |
| wavelet-LLH_gldm_LowGrayLevelEmphasis_ADC | 0.02540077 |
| wavelet-LLH_glrlm_LongRunLowGrayLevelEmphasis_ADC | 0.04340902 |
| wavelet-LLH_glrlm_LowGrayLevelRunEmphasis_ADC | 0.02335327 |
| wavelet-LLH_glszm_LowGrayLevelZoneEmphasis_ADC | 0.01977826 |
| wavelet-LLH_glszm_ZoneEntropy_ADC | 0.01263674 |
| wavelet-LHH_firstorder_Maximum_ADC | 0.00080344 |
| wavelet-LHH_firstorder_Minimum_ADC | 0.00838136 |
| wavelet-LHH_firstorder_Range_ADC | 0.00211571 |
| wavelet-LHH_glcm_Autocorrelation_ADC | 0.00206082 |
| wavelet-LHH_glcm_ClusterProminence_ADC | 0.00572532 |
| wavelet-LHH_glcm_JointAverage_ADC | 0.01791472 |
| wavelet-LHH_glcm_SumAverage_ADC | 0.01791471 |
| wavelet-LHH_gldm_HighGrayLevelEmphasis_ADC | 0.00208177 |
| wavelet-LHH_gldm_LargeDependenceHighGrayLevelEmphasis_ADC | 0.00173527 |
| wavelet-LHH_gldm_SmallDependenceHighGrayLevelEmphasis_ADC | 0.00545788 |
| wavelet-LHH_glrlm_HighGrayLevelRunEmphasis_ADC | 0.00209295 |
| wavelet-LHH_glrlm_LongRunHighGrayLevelEmphasis_ADC | 0.00178113 |
| wavelet-LHH_glrlm_ShortRunHighGrayLevelEmphasis_ADC | 0.00291352 |
| wavelet-LHH_glszm_GrayLevelVariance_ADC | 0.01337171 |
| wavelet-LHH_glszm_HighGrayLevelZoneEmphasis_ADC | 0.0018522 |
| wavelet-LHH_glszm_SmallAreaHighGrayLevelEmphasis_ADC | 0.00112495 |
| wavelet-LHH_ngtdm_Complexity_ADC | 0.00215551 |
| wavelet-LHH_ngtdm_Contrast_ADC | 0.0415301 |
| wavelet-HLL_firstorder_10Percentile_ADC | 0.01115278 |
| wavelet-HLL_firstorder_90Percentile_ADC | 0.01222974 |
| wavelet-HLL_firstorder_InterquartileRange_ADC | 0.00886168 |
| wavelet-HLL_firstorder_MeanAbsoluteDeviation_ADC | 0.01146276 |
| wavelet-HLL_firstorder_RobustMeanAbsoluteDeviation_ADC | 0.00925774 |
| wavelet-HLL_firstorder_RootMeanSquared_ADC | 0.01552685 |
| wavelet-HLL_firstorder_Variance_ADC | 0.01671941 |
| wavelet-HLL_glcm_ClusterTendency_ADC | 0.01162459 |
| wavelet-HLL_glcm_Contrast_ADC | 0.03329569 |
| wavelet-HLL_glcm_DifferenceAverage_ADC | 0.02943451 |
| wavelet-HLL_glcm_DifferenceVariance_ADC | 0.04889482 |
| wavelet-HLL_glcm_InverseVariance_ADC | 0.0190997 |
| wavelet-HLL_glcm_SumSquares_ADC | 0.01563456 |
| wavelet-HLL_gldm_DependenceNonUniformityNormalized_ADC | 0.04439226 |
| wavelet-HLL_gldm_GrayLevelVariance_ADC | 0.01660051 |
| wavelet-HLL_gldm_SmallDependenceEmphasis_ADC | 0.03190907 |
| wavelet-HLL_glrlm_GrayLevelVariance_ADC | 0.01760169 |
| wavelet-HLL_glrlm_RunEntropy_ADC | 0.0116707 |
| wavelet-HLL_glszm_GrayLevelVariance_ADC | 0.0368278 |
| wavelet-HLL_glszm_ZonePercentage_ADC | 0.02764591 |
| wavelet-HLL_ngtdm_Contrast_ADC | 0.03289154 |
| wavelet-HLL_ngtdm_Strength_ADC | 0.02008828 |
| wavelet-HLH_glcm_Idmn_ADC | 0.02539984 |
| wavelet-HLH_glcm_Idn_ADC | 0.01757056 |
| wavelet-HLH_glrlm_ShortRunLowGrayLevelEmphasis_ADC | 0.04320032 |
| wavelet-HLH_ngtdm_Contrast_ADC | 0.0309606 |
| wavelet-HHH_firstorder_Kurtosis_ADC | 0.00582188 |
| wavelet-HHH_firstorder_Maximum_ADC | 0.01246285 |
| wavelet-HHH_firstorder_Minimum_ADC | 0.00673618 |
| wavelet-HHH_firstorder_Range_ADC | 0.00820502 |
| wavelet-HHH_glcm_Autocorrelation_ADC | 0.00462952 |
| wavelet-HHH_glcm_ClusterProminence_ADC | 0.00532826 |
| wavelet-HHH_glcm_DifferenceVariance_ADC | 0.0087173 |
| wavelet-HHH_glcm_MaximumProbability_ADC | 0.01288465 |
| wavelet-HHH_gldm_HighGrayLevelEmphasis_ADC | 0.00462079 |
| wavelet-HHH_gldm_LargeDependenceHighGrayLevelEmphasis_ADC | 0.00524794 |
| wavelet-HHH_gldm_SmallDependenceHighGrayLevelEmphasis_ADC | 0.00528355 |
| wavelet-HHH_glrlm_HighGrayLevelRunEmphasis_ADC | 0.00462936 |
| wavelet-HHH_glrlm_LongRunHighGrayLevelEmphasis_ADC | 0.00567064 |
| wavelet-HHH_glrlm_ShortRunHighGrayLevelEmphasis_ADC | 0.00433732 |
| wavelet-HHH_ngtdm_Complexity_ADC | 0.00291635 |
| wavelet-LLL_firstorder_10Percentile_ADC | 0.02234982 |
| wavelet-LLL_firstorder_90Percentile_ADC | 0.00624807 |
| wavelet-LLL_firstorder_InterquartileRange_ADC | 0.03660876 |
| wavelet-LLL_firstorder_Mean_ADC | 0.00411196 |
| wavelet-LLL_firstorder_Median_ADC | 0.00338205 |
| wavelet-LLL_firstorder_RobustMeanAbsoluteDeviation_ADC | 0.04220329 |
| wavelet-LLL_firstorder_RootMeanSquared_ADC | 0.00430652 |
| wavelet-LLL_glcm_ClusterTendency_ADC | 0.01803977 |
| wavelet-LLL_glcm_Contrast_ADC | 0.02475401 |
| wavelet-LLL_glcm_DifferenceAverage_ADC | 0.01557141 |
| wavelet-LLL_glcm_SumSquares_ADC | 0.01325851 |
| wavelet-LLL_gldm_DependenceNonUniformityNormalized_ADC | 0.01315401 |
| wavelet-LLL_gldm_SmallDependenceEmphasis_ADC | 0.03305175 |
| wavelet-LLL_glszm_SizeZoneNonUniformityNormalized_ADC | 0.02836058 |
| wavelet-LLL_ngtdm_Contrast_ADC | 0.02403276 |
| wavelet-LLL_ngtdm_Strength_ADC | 0.02038633 |
